# Beyond natural amino acids: extending immunogenicity risk assessment to modified peptides through chemical feature encoding. A case study on citrullination

**DOI:** 10.3389/fimmu.2026.1869623

**Published:** 2026-09-09

**Authors:** Maria Cairoli, Morten Nielsen, Catherine Betts, Olga Obrezanova, Leonardo De Maria

**Affiliations:** 1 Discovery Computation and AI, Hit Discovery, Discovery Sciences, Biopharmaceuticals R&D, AstraZeneca, Gothenburg, Sweden; 2 Department of Health Technology, Technical University of Denmark, Lyngby, Denmark; 3 Immune Safety, Clinical Pharmacology and Safety Sciences, Biopharmaceuticals R&D, AstraZeneca, Cambridge, United Kingdom; 4 Biologics Engineering, Oncology R&D, AstraZeneca, Cambridge, United Kingdom

**Keywords:** chemical fingerprints, immunogenicity risk assessment, machine learning, MHC class II, non-natural amino acids, post-translational modifications

## Abstract

**Introduction:**

Peptide therapeutics are increasingly explored to treat challenging diseases, but immunogenicity risks limit their clinical success. *In silico* tools enable immunogenicity screening through prediction of peptide-MHCII binding, yet current methods do not account for chemical properties of non-natural amino acids routinely incorporated to improve peptide properties.

**Methods:**

Here, we present a machine learning approach combining chemical fingerprints with sequence information to predict MHC class II binding for peptides including both natural (NAA) and non-natural (NNAA) amino acids. We evaluate peptide-level fingerprints against residue-level fingerprints (direct-encoding and similarity-based fingerprints) that preserve positional information while encoding chemical diversity, for a total of 31 different representations.

**Results:**

Peptide-level fingerprints showed poor performance due to loss of positional information, while residue-level fingerprints matched the performance of sequence-based encodings (BLOSUM62 and one-hot) for NAA-based peptides while accurately identifying binding cores and motifs. Considering citrullinated peptides as a case study, we observed similar linear correlation performance across encoding strategies, with residue-level fingerprints showing marginal improvements in quantitative prediction accuracy. Citrulline was rarely found at canonical anchor positions, suggesting that the advantage of explicit chemical encoding may be more pronounced for modifications occurring at positions critical for binding.

**Discussion:**

The proposed framework allows for inclusion of diverse modifications, including synthetic amino acids, and is compatible with existing pan-allele architectures. While the full advantage of explicit chemical encoding remains to be demonstrated, this framework is designed to capture these effects as experimental data becomes available, supporting immunogenicity risk assessment for emerging peptide therapeutics.

## 1 Introduction

Peptide therapeutics are increasingly relevant in the global pharmaceutical industry, playing a key role in treating some of the most challenging diseases, including diabetes, cancer and multiple sclerosis (1–3). Their intermediate size between small molecules and biologics confers advantageous properties such as high target specificity and minimal off-target effects (4). However, the clinical efficacy of such therapeutics is often limited by suboptimal properties, including short *in vivo* half-lives, poor oral bioavailability, limited permeation across biological barriers, and immunogenicity (2, 5). To improve these properties while preserving biological activity, medicinal chemists increasingly incorporate non-natural amino acids (NNAAs) beyond the 20 natural building blocks (4, 6, 7). However, despite these advances, the extent to which NNAAs affect immunogenicity remains mostly unknown (8), representing a critical concern for the success of clinical trials (9).

Immunogenicity refers to the ability of proteins or peptide biologics to elicit an unwanted immune response (10). Such response occurs when peptides are presented on major histocompatibility complex (MHC) molecules to T cells, leading to their activation, proliferation and differentiation into effector cells that can recognize and eliminate the antigen (11). In this process, peptide-MHC (pMHC) binding is a key factor determining which peptides are presented to T cells and will ultimately trigger an immune response (11). Predicting the binding to MHC class II molecules helps identify potential T cell epitopes that can drive the T-cell dependent B cell response, which is central to anti-drug antibody (ADA) production.

*In silico* methods have been developed to predict pMHCII binding and recognize potentially immunogenic peptides (12–14), and have been demonstrated powerful for preclinical immunogenicity assessment before costly clinical trials (15). These tools simultaneously address peptide alignment and binding motif characterization due to the open-ended MHC class II binding groove, which accommodates variable-length peptides and allows flanking regions to influence binding affinity (11). The introduction of NNAAs could affect these binding interactions and potentially trigger T cell activation (8, 9). However, current prediction algorithms are primarily trained on the 20 natural amino acids (NAAs), and may not accurately predict binding for NNAA-based peptides (9). Despite models encoding NNAAs having been proposed (9, 16), they fail to capture the chemical diversity of NNAAs. For instance, Alvarez et al. used sparse encoding to model phosphorylated peptides in NetMHCIIphosPan (16), while Mattei et al. encoded NNAAs with a neutral place holder ‘X’, supporting only one modification type (9). Excluding explicit chemical information limits the ability to distinguish among chemically diverse non-natural residues, potentially hampering accurate binding affinity prediction and preventing the inclusion of a broader range of synthetic modifications that medicinal chemists routinely employ in modern peptide therapeutic design.

Chemical fingerprints such as Extended Connectivity Fingerprints (ECFP) (17) and MinHashed Atom-Pair Chiral (MAPC) (18) are increasingly used in machine learning applications to describe peptides (19–21). These are derived from Simplified Molecular Input Line Entry System (SMILES) representation, which enables the description of molecular structures for peptides including both NAAs and NNAAs. To the best of our knowledge, however, these fingerprints have not yet been tested on peptide-MHC class II binding prediction. Unlike sequence-based encodings, canonical fingerprint applications encode entire peptides as unified molecular entities, which may not explicitly preserve the positional arrangement of amino acids critical for binding core identification.

Here, we evaluate the effectiveness of chemical fingerprints as alternative molecular representations for *in silico* pMHCII prediction against sequence-based encodings. We compare fingerprints that encode peptides as single molecular entities and test two representations that combine chemical diversity with positional information. We focus on single-allele models to isolate the effects of molecular representation from pan-allele learning. We benchmark these approaches against established sequence-based encodings, validate performance on peptides including NAAs across MHC class II and I alleles, and ultimately assess performance on available NNAA-based peptides, which in this study include citrullinated peptides. The evaluated representations operate on the same principle as substitution matrices, making them compatible with pan-allele prediction methods such as NetMHCIIpan. By addressing a critical gap in immunoinformatics, this work establishes a flexible, chemistry-informed framework for immunogenicity risk assessment across the large chemical space of modern peptide therapeutics.

## 2 Methods

### 2.1 Data sets

#### 2.1.1 Peptide-MHC class II binding affinity data sets

We retrieved binding affinity data for peptide sequences from the Immune Epitope Database (IEDB, www.iedb.org) (22). For peptides including NAAs, we employed the data set curated by Nielsen et al. (13), encompassing binding affinity data for several alleles (**Table 1**). We retrieved NNAA-based peptides from the IEDB database, retaining those having quantitative binding affinity data (IC50) and clearly defined modification. This allowed retaining 225 citrullinated peptides for DRB1*01:01 and 283 citrullinated peptides for DRB1*04:01. We merged citrullinated peptides with NAA-based peptides following the ‘Hobohm1’ (23) inspired algorithm proposed by Nielsen et al. (12) to minimize the overlap between training and evaluation data. This algorithm creates non-redundant peptide clusters by grouping sequences that share identical 9-mer overlaps, then splits these clusters into five cross-validation subsets to minimize sequence redundancy between training and test data (12). We tokenized peptide sequences at the residue level, with NAAs represented by single-letter codes and NNAAs enclosed in square brackets (e.g., [Cit] for citrulline), using a pattern-matching algorithm to identify and extract tokens from peptide sequences. We applied this tokenization scheme consistently across all algorithms.

**Table 1:** Details of training data sets.

Data set	Allele	#Peptides	Sequence length (avg ± std)	#Citrullinated peptides
pMHCII (NAA-based peptides)	DRB1*01:01	5166	15.1 ± 1.2	–
	DRB1*04:01	1024	15.6 ± 2.5	–
	DRB1*03:01	1020	16.3 ± 2.6	–
	DRB1*11:01	950	15.5 ± 2.4	–
	DRB1*15:01	934	15.7 ± 2.2	–
	DRB5*01:01	924	15.5 ± 2.3	–
	DRB1*07:01	853	15.7 ± 2.4	–
	DRB1*04:04	663	15.3 ± 2.1	–
	DRB1*04:05	630	15.4 ± 1.8	–
	DRB3*01:01	549	15.6 ± 2.3	–
	DRB1*09:01	530	15.2 ± 1.4	–
	DRB1*13:02	498	15.6 ± 2.1	–
	DRB4*01:01	446	15.9 ± 2.3	–
	DRB1*08:02	420	15.4 ± 1.7	–
pMHCII (NAA- and NNAA-based peptides)	DRB1*01:01	5391	15.1 ± 1.1	225
	DRB1*04:01	1307	15.5 ± 2.3	283
pMHCI (NAA-based peptides)	HLA-A*03:01	5694	9	–
	HLA-A*11:01	4733	9	–
	HLA-B*07:02	4523	9	–
	HLA-A*02:03	4451	9	–
	HLA-B*15:01	4186	9	–

pMHCII, pMHCI: peptide-MHC class II and I binding; NAA: natural amino acid; NNAA: non-natural amino acid.

The binding affinity score was computed using Equation (1).

(1) $$ba_{score}=1-\frac{\log(ba)}{log(50000)}$$

where *ba* is the binding affinity (IC50) measured in nM (13). ba_score_ values lower than 0 and higher than 1 were clipped to [0,1] range to avoid bias. Note that the actual binding affinity values (IC50) can be directly recovered from this score, except when they fall outside the range [1, 50,000] nM, though this range covers the practically relevant interval for binding affinity measurements. **Table 1** reports details on these data sets.

#### 2.1.2 Peptide-MHC class I binding affinity data set

We considered the data set curated by Reynisson et al. (14) to retrieve quantitative binding affinity (*ba*) data for MHC class I alleles. We retained only peptides with a length equal to 9 amino acids, for five alleles encompassing both A and B allotypes: HLA-A*03:01, HLA-A*11:01, HLA-B*07:02, HLA-A*02:03, HLA-B*15:01 (**Table 1**). Data were clustered in five folds before prediction using the ‘Hobohm1’ inspired algorithm described above.

#### 2.1.3 Data set from human proteome

Predicting random sequences can be useful to determining a baseline or background distribution against which the conservation of the amino acid patterns in immunogenic peptides can be established. For NAA-based peptides, we extracted 20,420 reviewed (Swiss-Prot) sequences from the UniProt Knowledgebase (UniProtKB; https://www.uniprot.org/) (24), considering ‘Human’ as organism. We selected sequences that only include the 20 NAAs, which were then split into sequences of 9-mers and 15-mers for MHC class I and class II alleles, obtaining 10,398,028 and 10,584,973 sequences, respectively. For each MHC class, we randomly sampled 500,000 sequences ultimately employed for prediction.

#### 2.1.4 Citrullinated peptides data set

We extracted citrullinated peptides from the data set published by Rebak et al. (25) for binding motif evaluation of citrulline, as it encompasses 14,056 citrullination sites within 4,008 proteins (25). We selected only sequences containing citrullinated sites, excluding those including other modifications. Sequences longer than 15 amino acids were split in 15-mers. We excluded duplicates, sequences shorter than 9 amino acids and not containing any modification, obtaining a total of 43,894 sequences considered for prediction.

### 2.2 Molecular representations

We evaluated three chemical fingerprint-based approaches to encode peptides: peptide-level, direct-encoding, and similarity-based fingerprints. These were benchmarked against established sequence-based encoding methods for binding affinity prediction and binding motif recognition.

#### 2.2.1 Sequence-based encodings

##### 2.2.1.1 BLOSUM matrix

BLOSUM (block substitution matrix) (26) matrices are the state-of-the-art in immunoinformatics to encode amino acid sequences. These matrices are based on local alignments of conserved protein regions and are built by calculating the log-odds scores for amino acid substitutions observed in blocks of aligned sequences from the Blocks Database (27). Each BLOSUM matrix is derived from sequence alignments clustered at different percent identity thresholds, for the 20 NAAs. In this study we consider BLOSUM62, which uses sequences with no more than 62% identity.

##### 2.2.1.2 One-hot encoding

This method represents each amino acid as a unique binary vector of length 20 (for NAAs), where a single position is set to 1 and all other positions are 0. This encoding captures only categorical information without incorporating physicochemical properties or evolutionary relationships.

##### 2.2.1.3 NNAAIndex

The Index of Natural and Non-Natural Amino Acids (NNAAIndex) (28) is a structural matrix that characterizes 155 properties of 615 amino acids, including both NAAs and NNAAs. These properties are represented by six features that encompass geometric characteristics for the considered amino acids: H-bond, connectivity, accessible surface area, integy moments index, volume and shape (28). While this representation encodes chemical information, it is limited to a fixed vocabulary of pre-characterized amino acids and uses sequence information. We therefore categorize it as sequence-based encoding in this manuscript.

#### 2.2.2 Peptide-level fingerprints

We evaluated 10 peptide-level chemical fingerprints (**Table 2**) representing different classes (29), each capturing diverse chemical properties. These fingerprints encode the entire peptide as a single molecular entity.

**Table 2:** Details of peptide-level chemical fingerprints and sequence-based encodings.

Molecular repr.	Category	Level	Fingerprint class	Type	Size	Source	Chemical information (y/n)	Positional information (y/n)	Additional parameters
Extended Connectivity (ECFP)	Chemical FP	Atom	Circular	Binary	2048	RDKIT	y	n	Radius=2 (default),8
Functional Class (FCFP)	Chemical FP	Atom	Circular	Binary	2048	RDKIT	y	n	Radius=3
Topological Torsion (TT)	Chemical FP	Atom	Path	Count	2048	RDKIT	y	n	torsionAtomCount=4
Atom Pair (AP)	Chemical FP	Atom	Path	Count	2048	RDKIT	y	n	
AVALON	Chemical FP	Atom	Path	Count	1024	RDKIT	y	n	
RDKIT	Chemical FP	Atom	Path	Binary	2048	RDKIT	y	n	maxPath=7, minPath=1
MACCS	Chemical FP	Atom	Sub- structure	Binary	166	RDKIT	y	n	
MinHashed Atom-Pair Chiral (MAPC)	Chemical FP	Atom	String	Categorical	2048	Orsi et al. (18)	y	n	Radius=2
PepFuNN	Chemical FP	Amino acid		Binary	1024	Ochoa et al. (30)	y	n	Radius=2
AmorProt	Chemical FP	Amino acid		Binary	3238	Lee et al. (31)	y	n	A=10, W = 10, R = 0.85
BLOSUM	Sequence	Amino acid		Real-valued	N×20	Henikoff et al. (26)	n	y	Threshold= 62%
One-hot	Sequence	Amino acid		Binary	N×20		n	y	
NNAAIndex	Sequence	Amino acid		Real-valued	N×6	Liang et al. (28)	Only available amino acids	y	

Positional information refers to the position of the amino acid residues, and N is the number of amino acids in the peptide sequence.

##### 2.2.2.1 Circular fingerprints

Circular fingerprints describe the local chemical environment around each atom in a molecule up to a specific radius (number of bonds), encoding atom types, bond types and connectivity patterns (17). These features are hashed and aggregated into fixed-length binary or count vectors. We evaluated Extended Connectivity Fingerprints (ECFP) (17) and Functional Class Fingerprints (FCFP) as representatives of this class.

##### 2.2.2.2 Path-based fingerprints

Path-based fingerprints enumerate the connected atom paths in the molecule and hash them into fixed-length binary or count vectors (29). From this class, we evaluated RDKIT, AVALON, Topological Torsion and Atom Pair fingerprints.

##### 2.2.2.3 Substructure-based fingerprints

Substructure-based fingerprints encode the presence or absence of predefined structural fragments, including functional groups and specific atom environments. We evaluated MACCS keys (32), which use 166 predefined fragments.

##### 2.2.2.4 String-based fingerprints

String-based fingerprints use SMILES string representations as input and apply text-based hashing methods to encode molecular structure (29). We selected MinHashed Atom-Pair Chiral (MAPC) fingerprints, which compute MinHashes from character strings containing SMILES representations of all circular substructure pairs and their topological distances (18).

For all fingerprints, we adopted software-default parameters (**Table 2**). Given the frequent use of Extended Connectivity Fingerprints ECFP16 (Radius = 8) for peptides (20), this variant was also evaluated. We additionally considered fingerprints specifically developed for peptide and protein encoding: PepFuNN and AmorProt.

##### 2.2.2.5 PepFunNN

PepFuNN (30) is an open-source Novo Nordisk package that builds fingerprints based on monomer composition by converting peptides into graphs with monomers as nodes, calculating tokens from graph-derived fragments, and generating binary fingerprints.

##### 2.2.2.6 AmorProt

AmorProt (31) is a protein sequence representation method combining molecular fingerprints of constituent amino acids into a unified peptide fingerprint. Individual amino acid fingerprints (MACCS, ECFP4, ECFP6, and RDKIT) are multiplied by smoothed trigonometric functions, summed column-wise, and normalized to maximum values (31).

#### 2.2.3 Direct-encoding fingerprints (residue-level)

The described representations generally capture either chemical or positional information but fail to encompass both properties simultaneously (**Table 2**). To address this limitation, we tested two types of residue-level fingerprints that preserve both chemical and positional properties.

Each amino acid, irrespective of whether it is natural or non-natural, can be represented by its SMILES notation and subsequently encoded as a chemical fingerprint. We construct a matrix **F** with dimensions M × N, where each row represents an N-dimensional chemical fingerprint for one of M amino acids in the sequence. By replacing each amino acid with its corresponding fingerprint, we create a position-specific representation that preserves sequence order while incorporating chemical information. Since fingerprints describe individual amino acids, we considered a fingerprint length of N = 256 for all representations, except for MACCS which has a fixed length of N = 166 and AmorProt (N = 934).

#### 2.2.4 Similarity-based fingerprints (residue-level)

To further explore this concept, we constructed similarity matrices **S** with dimensions M × M, where each entry represents the Tanimoto similarity coefficient between amino acid pairs based on their chemical fingerprints. By encoding pairwise amino acid relationships rather than individual properties, this approach conceptually resembles substitution matrices like BLOSUM and was therefore evaluated alongside direct-encoding fingerprints.

Direct-encoding and similarity-based fingerprints were independently calculated for each considered chemical fingerprint (**Table 2**), yielding 20 distinct matrices for performance comparison. According to the considered fingerprint, we computed Tanimoto similarity as described in Ref (29). Throughout this work, we denote direct-encoding fingerprints as **F**_fps_ and similarity-based fingerprints as **S**_fps_ where ‘*fps’* indicates the underlying chemical fingerprint (e.g., **F**_ECFP16_, **S**_ECFP16_). We excluded PepFuNN from these representations as it considers the entire peptide for fingerprint calculation. Prior to fingerprint conversion, SMILES strings were canonicalized using RDKit applying charge neutralization and preserving stereochemical information. For consistency across all encoding methods, the BLOSUM62 matrix, one-hot encoding matrix, and similarity matrix **S** are all of dimensions 22 × 22, encompassing the 20 NAAs, citrulline and a placeholder token ‘X’ for unknown residues. For BLOSUM62 and one-hot encoding, we assigned citrulline and ‘X’ zero scores for all pairwise comparisons. The direct-encoding fingerprints **F** have dimensions 22 × 256 for all fingerprints except for MACCS (22 × 166) and AmorProt (22 × 934). The NNAAIndex matrix has dimensions 22 × 6. We assigned zero scores to ‘X’ for all the obtained matrices.

### 2.3 Neural network model

To test the different representations on NAA- and NNAA-based peptides, our machine learning framework builds upon the NNAlign model (13). This method allows predicting peptide binding affinity and extracting the optimal binding core of 9 amino acids. All possible 9-mer windows within each peptide are extracted and processed through a neural network to generate binding scores for each 9-mer. The model then selects the 9-mer with the highest predicted binding score as the predicted binding register for that peptide. This score is compared against the peptide binding affinity score, and the loss is backpropagated to update the network parameters. Through this process, the model learns to identify which 9-mer core within each peptide is most likely responsible for MHC binding. Flanking regions were also considered to capture both the binding register and its context, enabling the neural network to learn binding cores while accounting for the influence of neighboring residues on pMHCII interactions. For sequence-based encodings, direct-encoding and similarity-based fingerprints, we encoded flanking regions by calculating average scores for sequences surrounding each 9-mer window. The left flank extends backward from the window start, while the right flank extends forward from the window end, with zero-padding applied at sequence boundaries. For peptide-level fingerprints, each flanking region was directly converted to its own fingerprint.

We implemented the neural network model as a feed-forward multilayer perceptron (MLP) with two fully connected hidden layers, each followed by batch normalization (batch size = 32), dropout and rectified linear unit (ReLU) activation function for stabilized and regularized training, employing Adam with weight decay algorithm (33) for stochastic optimization. Considering that the binding affinity score ranges between 0 and 1, we added sigmoid activation to ensure that the output falls in this range. To reduce the potential impact of redundancy present in the binding data (13), we modified the network back-propagation so that the step size was divided by the binding core redundancy of the given peptide, as proposed by Nielsen et al. (13). During core selection we applied a burn-in phase of 15 epochs, in which position 1 was restricted to hydrophobic amino acids (I, L, V, M, F, Y, W). With limited data sizes, this step is typically required to guide a correct reconstruction of MHCII binding motifs (34, 35). This does not apply for the predictions considering MHC class I alleles and peptide-level fingerprints. We optimized hidden layer dimension, dropout rate, learning rate and weight decay using Optuna (36) (**Table 3**) considering 50 iterations and pruning. For the data sets including citrullinated peptides, an imbalance factor was further tuned during training to account for imbalance between NAA-based and citrullinated peptides.

**Table 3:** Search space of hyperparameters optimized with Optuna.

Hyperparameter	Search space
hidden layer dimension	[2, 8, 16, 32, 64]
dropout rate	[0.3, 0.8] (uniform float)
learning rate	[1^-5^,5^-3^] (log-uniform float)
weight decay	[1^-4^,1^-2^] (log-uniform float)
imbalance factor	[0, 10] with step 1 (integer)

As proposed by Nielsen et al. (13), we evaluated a neural network within a nested cross-validation and ensemble framework. We used five-fold outer cross-validation, holding out one fold for evaluation and using the remaining four folds for hyperparameter optimization via a nested four-fold cross-validation. For each Optuna trial, we trained models on three folds and validated on the fourth. Each model was run for 500 epochs, monitoring early stopping (minimum change = 10^-3^, patience = 10) on the validation fold to prevent overfitting. We ranked trials by the mean nested-fold MSE, and the top 5 trials were retained per outer iteration. Each retained trial contributed to its four nested-fold models, yielding an ensemble of up to 20 models per outer fold. We obtained ensemble predictions by averaging model outputs, and binding cores by majority vote across models.

#### 2.3.1 Mode of presentation

We adopted two different modes of presentation according to the considered molecular representation. For sequence-based encodings, direct-encoding and similarity-based fingerprints, we encoded each amino acid using the respective scores, producing concatenated feature vectors of dimension 9 × *d*, where *d* is the dimension of the input representation. These were concatenated with the average flanking features, yielding final feature vectors with dimension *(9 × d) + 2d*.

For peptide-level fingerprints, the computational cost of calculating fingerprints for each 9-mer window and flanking regions required pre-computation before model training. We processed peptide sequences using a sliding window to extract all possible 9-mer cores, converting each sequence into a single molecular fingerprint. We concatenated core fingerprints with flanking region fingerprints, yielding feature vectors of dimension 3*d*, where *d* is the fingerprint dimension (**Table 2**).

### 2.4 Performance evaluation

We used MSE for network training and best model selection. We evaluated model performances through regression lines and coefficient of determination R^2^, root mean square error (RMSE) and Pearson correlation coefficient (PCC). These metrics were computed on the aggregated out-of-fold predictions. In line with studies in immunogenic bioinformatics, we report PCC as main metric for multiple comparisons, reporting R^2^ performances in the **Supplementary Material**. R^2^ and RMSE are defined in Equations 2, 3.

(2) $$R^{2}=1-\;\frac{\sum_{i=1}^{n}{\left(y_{i}-{\hat{y}}_{i}\right)}^{2}}{\sum_{i=1}^{n}{\left(y_{i}-\bar{y}\right)}^{2}}$$

(3) $$RMSE=\;\sqrt{\frac{\sum_{i=1}^{n}{\left(y_{i}-{\hat{y}}_{i}\right)}^{2}}{n}}$$

where *n* is the number of samples, ${\hat{y}}_{i}$ is the predicted value of the *i*-th sample, $y_{i}$ is the corresponding measured value and $\bar{y}$ the mean measured value over *n* samples.

To test for statistically significant differences among molecular representations for NAA-based peptides, we compared each representation to BLOSUM62 using paired, per-sample squared error differences computed from out-of-fold predictions using a two-sided Wilcoxon signed-rank test. To account for multiple comparisons across representations within each allele, we controlled the false discovery rate at α = 0.05 using the Benjamini-Hochberg procedure, considering significant adjusted p-values < 0.05. For citrullinated peptides, we applied the same statistical testing procedure but using BLOSUM62 with citrulline substituted by arginine as the reference baseline, since arginine is the closest natural analogue to citrulline. The case where citrulline is substituted by an unknown residue ‘X’ is also reported for comparison, as it may represent a scenario more relevant to NNAAs for which a direct natural analogue is not available.

We performed all tests on aggregated out-of-fold predictions using the same cross-validation splits across representations. We calculated metric confidence intervals by bootstrapping the out-of-fold predictions with 10,000 resamples, reporting the 2.5^th^ and 97.5^th^ percentiles of the bootstrap distribution as error bars.

### 2.5 Sequence logos

We calculated sequence logos following the approach presented in Seq2Logo (37), considering Kullback-Leibler divergence, prior weights = 200, and sequence weighting based on a “Hobohm1” like algorithm using an identity threshold of 0.63. For the external test sets derived from the human proteome, sequence logos were constructed from the predicted binding cores of the top 1% strongest predicted binders. For cross-validated predictions, the number of peptides is relatively small (i.e., lower than 6,000): all the peptides predicted as binding (ba_score_ ≥ 0.5) were considered for sequence logos, after aggregating estimated binding cores from all out-of-fold predictions.

### 2.6 Evaluating learned binding cores

To evaluate the performance of our method in extracting binding cores, we compared it against two baseline approaches based on TEPITOPE (38), a widely-used benchmark for single-allele MHC class II prediction. The baselines consisted of i) TEPITOPE’s direct predictions, and ii) a two-stage approach where cores extracted from TEPITOPE serve as input to machine learning regressors (MLP, SVM, or XGBoost). This comparison considers BLOSUM62 as representation and allows evaluating whether cores learned during training by our algorithm match or exceed the performance achieved using TEPITOPE-derived cores. We evaluated all approaches on both binding affinity prediction accuracy and binding motifs derived from top-1% binders. Additional details regarding this comparison are reported in the **Supplementary Material** (**Supplementary Figures 12**, **S13**; **Supplementary Table 1**).

### 2.7 Evaluating the effect of encoding chemical information

To test for the importance of encoding chemical information for NNAAs we performed three complementary comparisons. First, we evaluated whether chemical fingerprints improve prediction accuracy when models are trained and tested on data sets that include citrullinated peptides. We trained the models using different encoding strategies: i) explicit encoding of citrulline, ii) substitution of citrulline with arginine, iii) substitution with ‘X’. Models were trained and evaluated on the same data sets under each encoding strategy, considering BLOSUM62 with arginine substitution as the primary baseline. To evaluate the occurrence of citrulline at binding cores, we analyzed the data set derived from Rebak et al. (25) and assessed the frequency of citrullinated residues across the top 1% strongest predicted binders for each encoding strategy.

Second, to assess model generalization to citrullinated peptides, we trained the models exclusively on NAA-based peptides and evaluated their predictions on citrullinated peptides. In this comparison, citrulline was encoded as-is for direct-encoding and similarity-based fingerprints, and as ‘X’ tokens or arginine for sequence-based encodings.

Third, to further evaluate generalization abilities and the impact of training data composition for direct-encoding and similarity-based fingerprints, we extended the second approach by systematically varying exposure to citrullinated peptides during training. Starting from the original data set including NAA-based and citrullinated peptides (**Table 1**), we performed 5-fold cross-validation where each fold served as a test set once. For each cross-validation split, we created five training configurations from the remaining four folds, each containing 0%, 25%, 50%, 75%, or 100% of available citrullinated peptides while retaining all NAA-based peptides, maintaining the ‘Hobohm1’ criterion across both NAA-based and citrullinated peptides to minimize sequence redundancy, as described above. We evaluated performances by aggregating predictions across all five test folds for each training configuration, separately calculating metrics for citrullinated peptides.

## 3 Results

### 3.1 Direct-encoding and similarity-based fingerprints match sequence encodings for NAA-based peptides

We first evaluated molecular representations based on two criteria: their ability to predict binding affinity scores and their accuracy in assigning binding cores for alleles with experimentally determined cores from 15 HLA-DR restricted peptides compiled from the Protein Data Bank available from Ref (13).

Overall, peptide-level fingerprints yielded significantly worse binding affinity predictions than BLOSUM62 (p<0.05), although some representations (e.g., Atom Pair fingerprints) performed comparatively better within this class (**Supplementary Figures 1**, **S2**). Importantly, most binding cores (80-100%) were incorrectly identified, even for representations specifically designed for peptides (**Supplementary Figure 3**). By aggregating chemical information across the entire peptide sequence, these representations lose the positional information required to identify anchor residues at defined positions. This limitation results in predictions lacking biological interpretability, posing potential challenges for downstream drug discovery applications.

**Figure 1:**
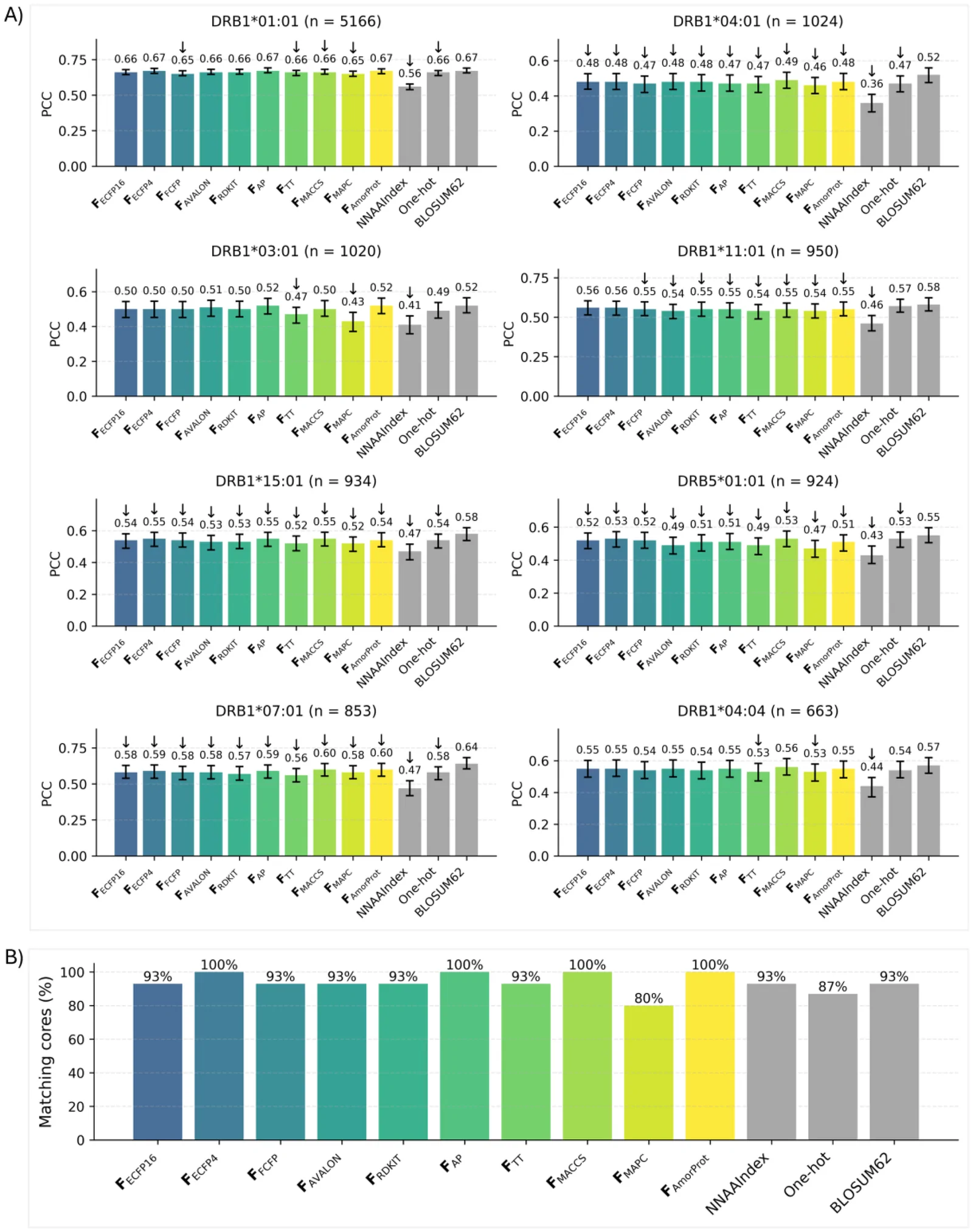
Performance of direct-encoding fingerprints for MHC class II binding prediction. **(A)** PCC values from out-of-fold predictions for NAA-based peptides binding to selected HLA-DR alleles. Each subplot shows results for one allele with sample size indicated in the title. Gray bars represent sequence-based encodings; colored bars represent direct-encoding fingerprints. Upward arrows indicate significantly better performance than BLOSUM62; downward arrows indicate significantly worse performance than BLOSUM62; absence of arrows indicates no significant difference from BLOSUM62 (paired Wilcoxon tests on per-peptide squared errors, FDR < 0.05, Benjamini-Hochberg correction for 12 pairwise comparisons per allele). Error bars report the 2.5^th^–97.5^th^ percentiles of the bootstrap distribution (10,000 resamples). Performances for remaining alleles and in terms of R^2^ are reported in **Supplementary Figures 4**, **S5**. **(B)** Percentage of matching predicted binding cores for each representation against reference for 15 HLA-DR restricted peptides compiled from the protein database, retrieved from Ref (13). 80%, 87% and 93% correspond to 12, 13 and 14 matching cores over 15, respectively. **Supplementary Figure 6** reports detailed information regarding core and peptide sequences.

**Figure 2:**
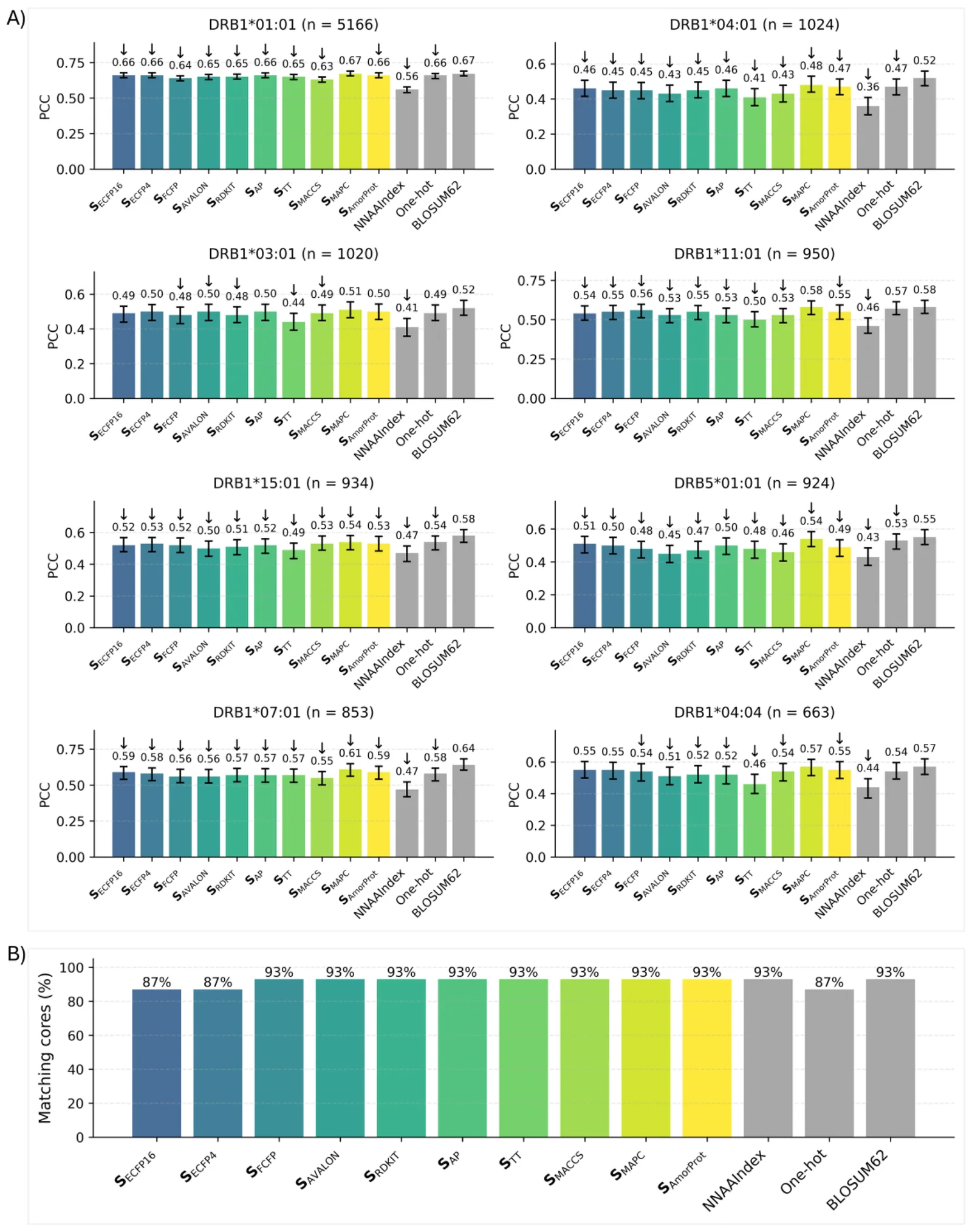
Performance of similarity-based fingerprints for MHC class II binding prediction. **(A)** PCC values from out-of-fold predictions for NAA-based peptides binding to selected HLA-DR alleles. Each subplot shows results for one allele with sample size indicated in the title. Gray bars represent sequence-based encodings; colored bars represent similarity-based fingerprints. Upward arrows indicate significantly better performance than BLOSUM62; downward arrows indicate significantly worse performance than BLOSUM62; absence of arrows indicates no significant difference from BLOSUM62 (paired Wilcoxon tests on per-peptide squared errors, FDR < 0.05, Benjamini-Hochberg correction for 12 pairwise comparisons per allele). Error bars report the 2.5^th^-97.5^th^ percentiles of the bootstrap distribution (10,000 resamples). Performances for the remaining alleles and in terms of R^2^ are reported in **Supplementary Figures 7**, **S8**. **(B)** Percentage of matching predicted binding cores for each representation against reference for 15 HLA-DR restricted peptides compiled from the protein database, retrieved from Ref (13). 87% and 93% correspond to 13 and 14 matching cores over 15, respectively. **Supplementary Figure 9** reports detailed information regarding core and peptide sequences.

In contrast, direct-encoding and similarity-based fingerprints correctly identified 80-100% of reference cores (12–14 out of 15 peptides), consistent with sequence-based encoding performance (**Figures 1**, **2B**). Binding affinity prediction performance varied across representations, with NNAAIndex showing the poorest performance overall. NNAAIndex comprises six representative features encompassing different amino acid properties (28); our results suggest that these features are insufficient to achieve performance comparable to BLOSUM62 and other representations for pMHCII prediction. Among direct-encoding fingerprints, ECFP4 (**F**_ECFP4_) and Atom Pair (**F**_AP_) demonstrated the best performance across alleles (**Figure 1A**; **Supplementary Figure 4**), while MAPC fingerprints (**S**_MAPC_) showed the best overall performance among similarity-based representations (**Figure 2A**; **Supplementary Figure 7**). This finding aligns with prior studies, where MAPC fingerprints performed well in several applications, and particularly in similarity tasks (19). The evaluated representations generally matched or outperformed TEPITOPE-derived predictions (**Supplementary Figure 12**), consistent with earlier findings (13) that integrated learning of binding cores during training yields competitive or better performance compared to pre-defined core extraction algorithms.

**Figure 3:**
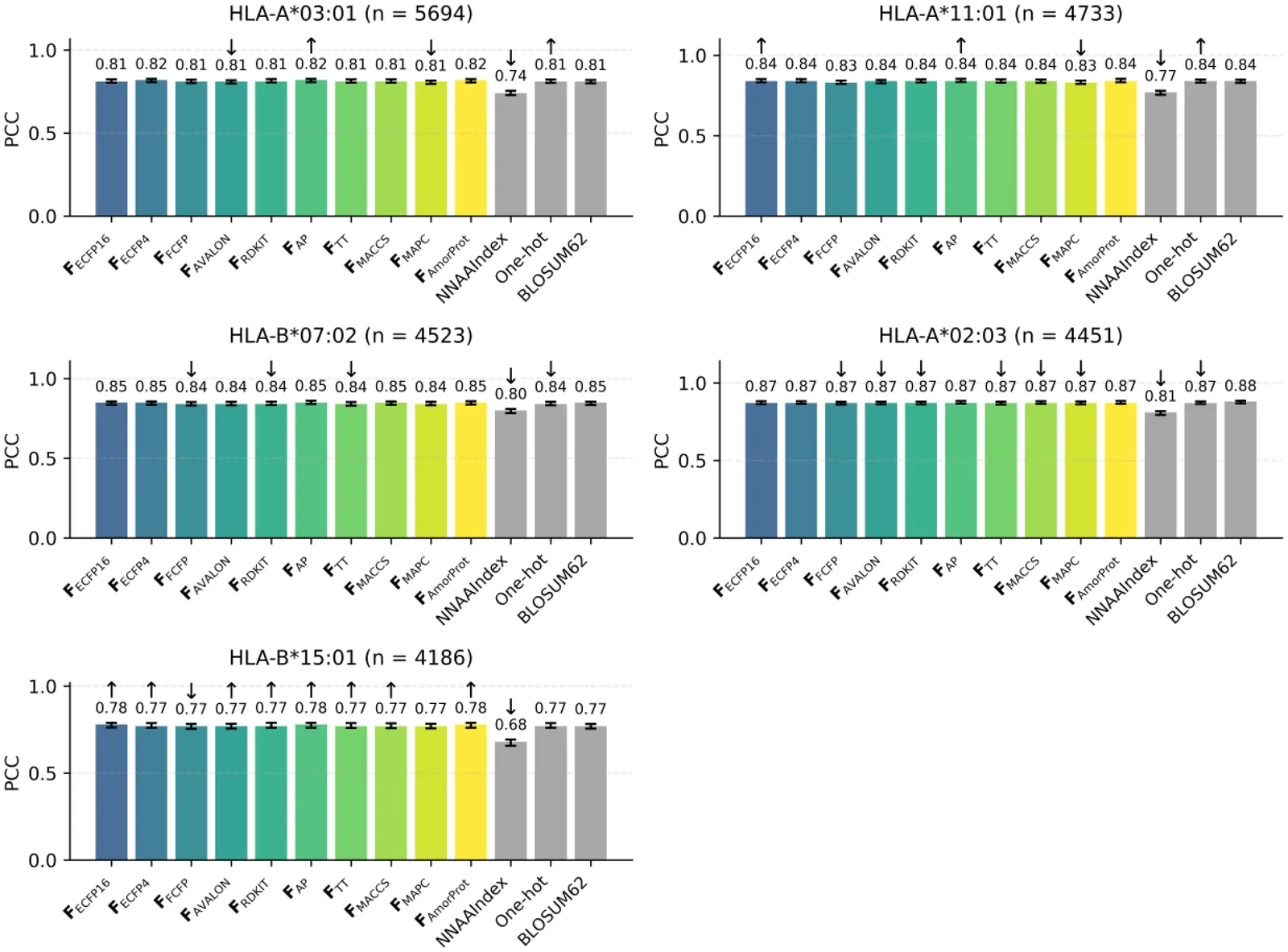
Performance of direct-encoding fingerprints for MHC class I prediction. PCC values from out-of-fold predictions for NAA-based peptides binding to selected HLA-A and HLA-B alleles. Each subplot shows results for one allele with sample size indicated in the title. Gray bars represent sequence-based encodings; colored bars represent direct-encoding fingerprints. Upward arrows indicate significantly better performance than BLOSUM62; downward arrows indicate significantly worse performance than BLOSUM62; absence of arrows indicates no significant difference from BLOSUM62 (paired Wilcoxon tests on per-peptide squared errors, FDR < 0.05, Benjamini-Hochberg correction for 12 pairwise comparisons per allele). Error bars report the 2.5^th^-97.5^th^ percentiles of the bootstrap distribution (10,000 resamples). Performances for similarity-based fingerprints and in terms of R^2^ are reported in **Supplementary Figures 14**–**S16**.

MHC class I binding predictions showed similar trends with improved overall performance compared to pMHCII binding prediction (**Figure 3**). MHC class I molecules have a closed binding groove that accommodates predominantly 9-mer peptides with specific residues at conserved positions fitting into deep hydrophobic pockets (39), rendering this prediction typically less challenging than pMHCII. Performance differences across representations are less pronounced than for MHC class II predictions, suggesting that the choice of the molecular representation may have less impact on performance for this task.

Based on these results, we selected four distinct encoding strategies for subsequent analyses. MAPC similarity-based fingerprints (**S**_MAPC_) demonstrated superior overall performance among similarity-based fingerprints and were selected alongside BLOSUM62 and one-hot encoding, two widely adopted sequence-based encoding methods in immunoinformatics. Among direct-encoding fingerprints, predicted binding scores for the best performing fingerprints showed high correlation (**Supplementary Figure 10**), indicating comparable predictive capability. We selected **F**_AP_ based on its overall better performance across alleles. These four representations employ different encoding strategies: **F**_AP_ and **S**_MAPC_ capture structural and chemical diversity, one-hot encoding provides categorical amino acid differentiation, and BLOSUM62 incorporates evolutionary substitution information. All four representations effectively preserve positional information.

### 3.2 Predicted binding motifs align with established patterns for NAA-based peptides

**Figure 4:**
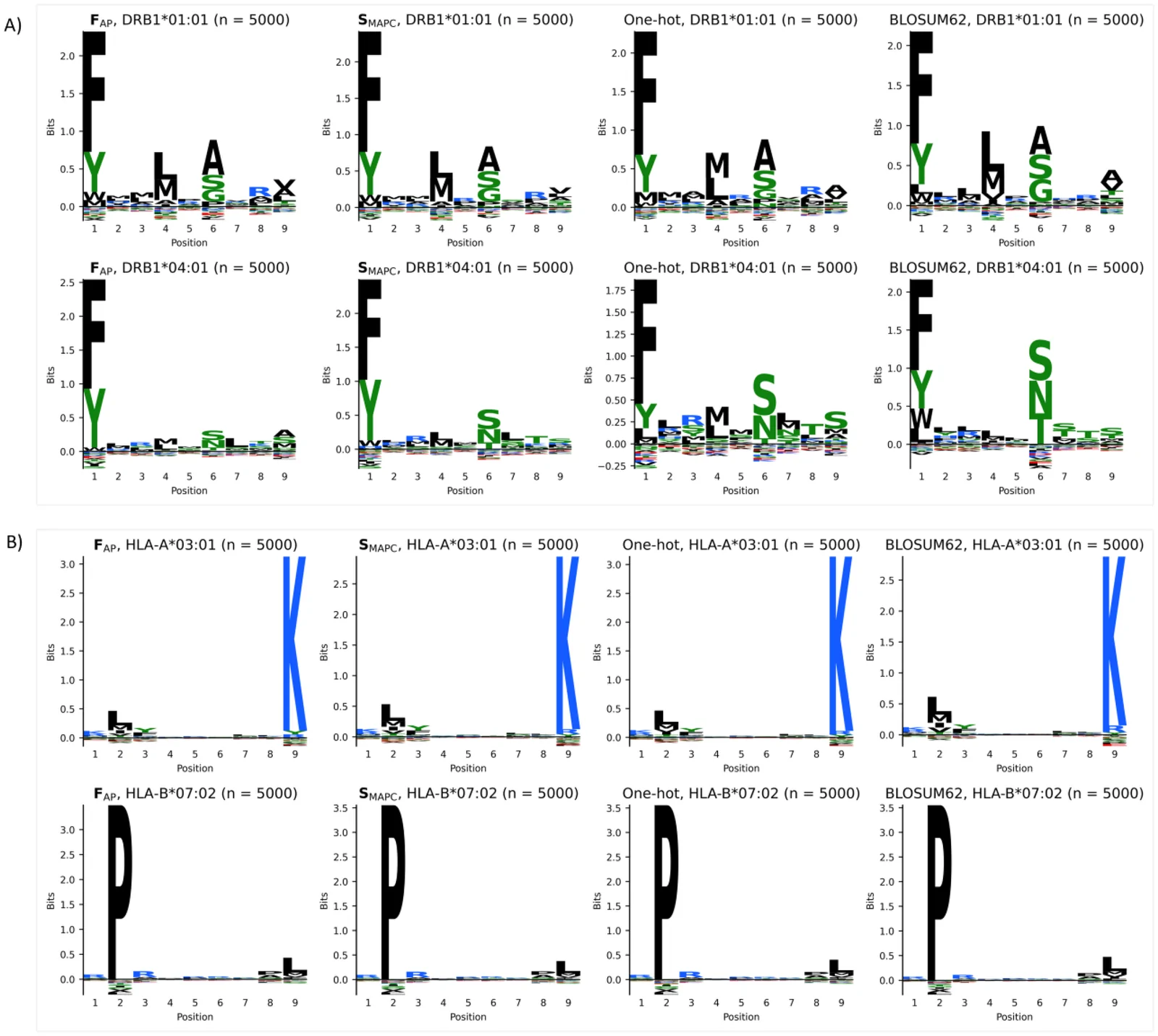
Predicted binding motifs for MHC class II and class I alleles. Sequence logos generated from the top 1% (n = 5,000) predicted binders among randomly sampled peptides from the UniProt human proteome for selected **(A)** MHC class II alleles and **(B)** MHC class I alleles, considering **F**_AP_, **S**_MAPC_, one-hot and BLOSUM62. Logos display amino acid frequencies at each position, with letter height proportional to information content. Sequence logos from out-of-fold predictions are provided in **Supplementary Figures 11**, **S17**.

Accurate binding motif prediction is essential for understanding pMHC interactions and immune response mechanisms. **Figure 4** presents predicted binding motifs for representative MHC class II and class I alleles, respectively. For MHC class II, common anchor residues are at positions P1, P4, P6, and P9, with hydrophobic amino acids predominating at P1 (40). The modelled binding motifs align well with the established patterns for peptides binding to HLA-DR alleles, characterized by large hydrophobic residues (predominantly phenylalanine and tyrosine) at position 1 and small uncharged residues at position 6 (41). This pattern is evident both in motifs derived from random epitope selection from the human proteome (**Figure 4A**) and out-of-fold predictions (**Supplementary Figure 11**) and remained consistent with TEPITOPE-derived methods (**Supplementary Figure 13**).

For MHC class I, primary anchor residues mainly occur at positions P2 and P9 of the binding 9-mer (40). **Figure 4B**, **Supplementary Figure 17** show that all selected representations successfully identify these anchor positions, both for peptides randomly sampled from the human proteome and out-of-fold predictions. HLA-A*03:01 favors leucine and lysine at positions 2 and 9, respectively, consistent with canonical HLA-A3 motifs (42). HLA-B*07:02 shows preference for proline at P2 and leucine at P9, aligning with established patterns (40, 43). These results demonstrate that residue-level fingerprints perform comparably to established encodings for NAAs. We next evaluate whether their explicit chemical representation provides an advantage for citrullinated peptides, where sequence-based encodings lack information about the modification.

### 3.3 Residue-level fingerprints show comparable performance to sequence encodings for citrullination at non-anchor positions

**Figure 5:**
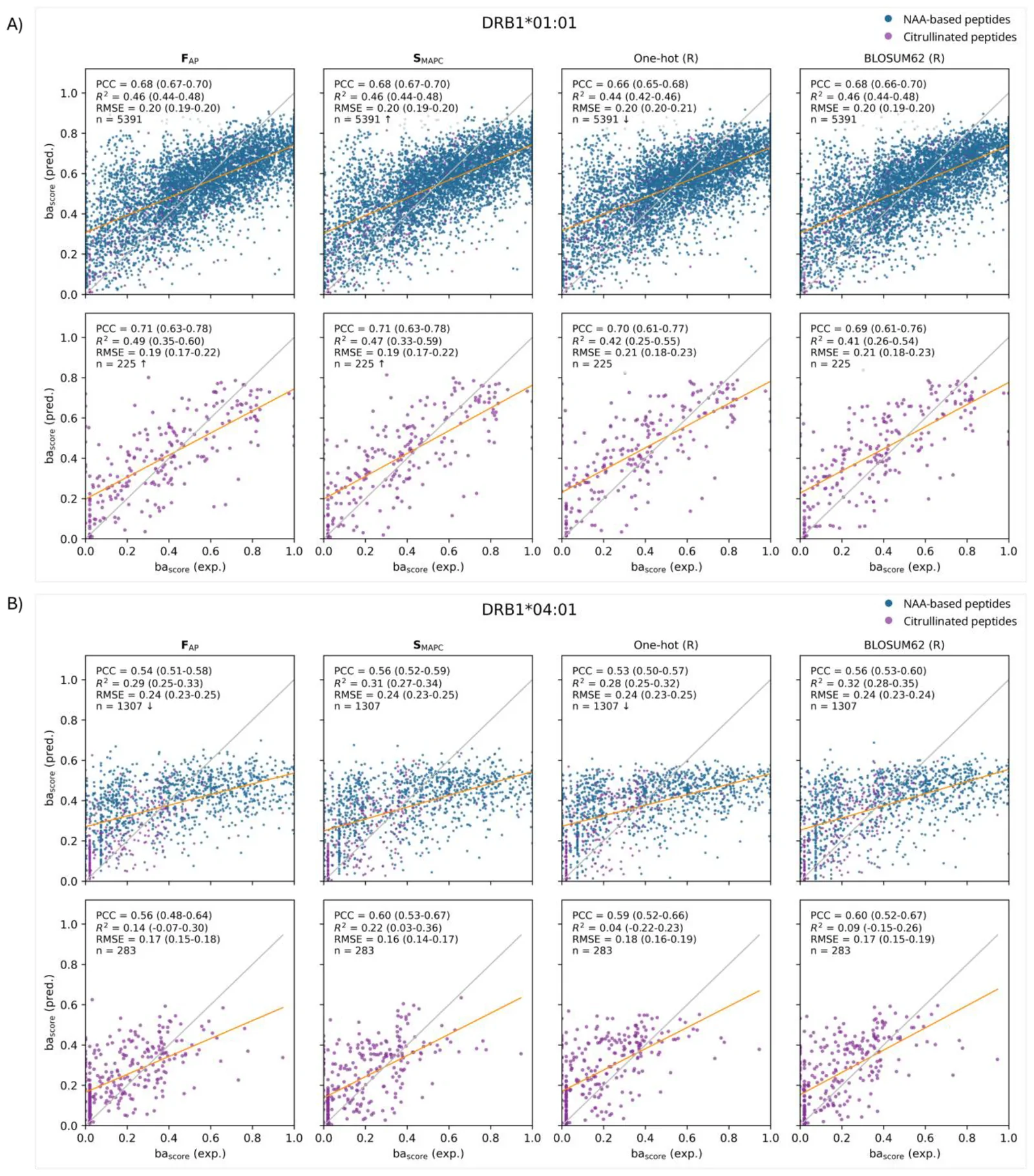
Prediction performance for citrullinated peptides with models trained on NAA-based and citrullinated peptides. Binding affinity predictions for **(A)** DRB1*01:01 and **(B)** DRB1*04:01 using models trained using both NAA-based and citrullinated peptides. In each subplot, the first row shows out-of-fold predictions on both NAA-based and citrullinated peptides, while the second row shows predictions on citrullinated peptides only. Blue dots: NAA-based peptides; purple dots: citrullinated peptides. Yellow line: fitted regression; gray line: identity line. Each panel shows results for four representations (**F**_AP_, **S**_APC_, one-hot, BLOSUM62) with PCC, R² and RMSE performance metrics, including the 2.5^th^ and 97.5^th^ percentiles of the bootstrap distribution (10,000 resamples). Upward arrows indicate significantly better performance than BLOSUM62 with citrulline encoded as arginine (R); downward arrows indicate significantly worse performance than BLOSUM62 (R); absence of arrows indicates no significant difference (paired Wilcoxon tests on per-peptide squared errors, FDR < 0.05, Benjamini-Hochberg correction for 3 pairwise comparisons per allele). n indicates the number of predicted samples.

**Figure 5** shows prediction performance for NAA-based and citrullinated peptides binding to DRB1*01:01 and DRB1*04:01 alleles using the four selected encoding strategies. For one-hot and BLOSUM62 encodings, citrulline was encoded as arginine as its closest natural analogue (**Figure 5**), or as an unknown residue ‘X’ (**Supplementary Figure 18**).

All encodings demonstrate comparable performance across both alleles, though predictions for DRB1*04:01 show reduced accuracy, likely due to limited training data and data set class imbalance, with fewer binders (ba_score_ ≥ 0.5) compared to non-binders, limiting generalization to binding peptides. PCC values remain relatively comparable across representations, indicating that all representations capture similar linear relationships between predicted and observed binding affinities. Residue-level fingerprints show marginal improvements in R^2^ values for citrullinated peptides when compared to one-hot and BLOSUM62 encodings when citrulline is encoded as arginine (**Figure 5**) or as an unknown residue ‘X’ (**Supplementary Figure 18**). This suggests a slight advantage for quantitative binding affinity predictions when modifications are explicitly represented.

**Figure 6:**
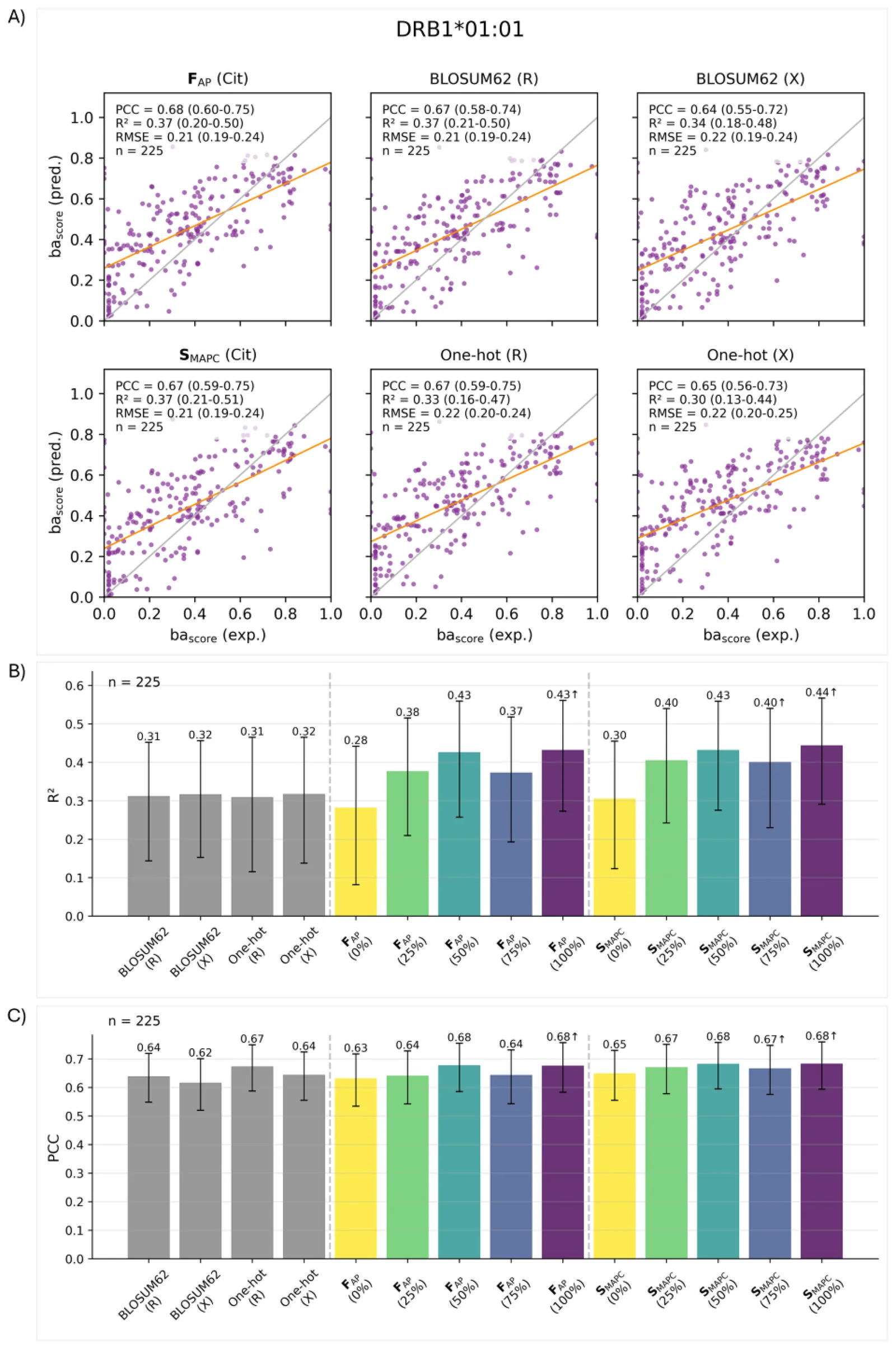
Impact of training data composition on prediction performance for citrullinated peptides (DRB1*01:01). **(A)** Prediction performance on citrullinated peptides using models trained exclusively on NAA-based peptides. Yellow line: fitted regression; gray line: identity line. Four representations are compared: **F**_AP_ and **S**_MAPC_ encode citrulline via chemical fingerprints; one-hot and BLOSUM62 encode citrulline as ‘X’ (zero vector) or ‘R’ (arginine). PCC, R² and RMSE performance metrics, including the 2.5^th^ and 97.5^th^ percentiles of the bootstrap distribution (10,000 resamples), are reported. **(B)** R² and **(C)** PCC as a function of citrullinated peptide proportion in training data (0%, 25%, 50%, 75%, 100%). Models were evaluated using 5-fold cross-validation. Gray bars: BLOSUM62 and one-hot encodings trained only on NAA-based peptides, with citrulline encoded as ‘X’ or ‘R’; colored bars: **F**_AP_ and **S**_MAPC_ performance at each training composition. Error bars report the 2.5^th^-97.5^th^ percentiles of the bootstrap distribution (10,000 resamples). Upward arrows indicate significantly better performance than BLOSUM62 with citrulline encoded as arginine (R); downward arrows indicate significantly worse performance than BLOSUM62 (R); absence of arrows indicates no significant difference (paired Wilcoxon tests on per-peptide squared errors, FDR < 0.05, Benjamini-Hochberg correction for 13 pairwise comparisons per allele). Results for DRB1*04:01 are reported in **Supplementary Figure 19**.

We further evaluated performance generalization by predicting citrullinated peptides using models trained exclusively on NAA-based peptides (**Figure 6A**; **Supplementary Figure 19A**). Without encoding citrullination information during training, residue-level fingerprints show no advantage over sequence-based encodings. However, when citrullinated peptides are progressively included during training, R² performance improves for direct-encoding and similarity-based fingerprints starting at 25-50% inclusion, while PCC remains relatively stable across all representations (**Figures 6B, C**; **Supplementary Figures 19B, C**). Overall, these results are consistent with the previous analysis, suggesting that the advantage of explicit chemical encoding for citrulline also depends on the availability of citrullinated peptides during training.

**Figure 7:**
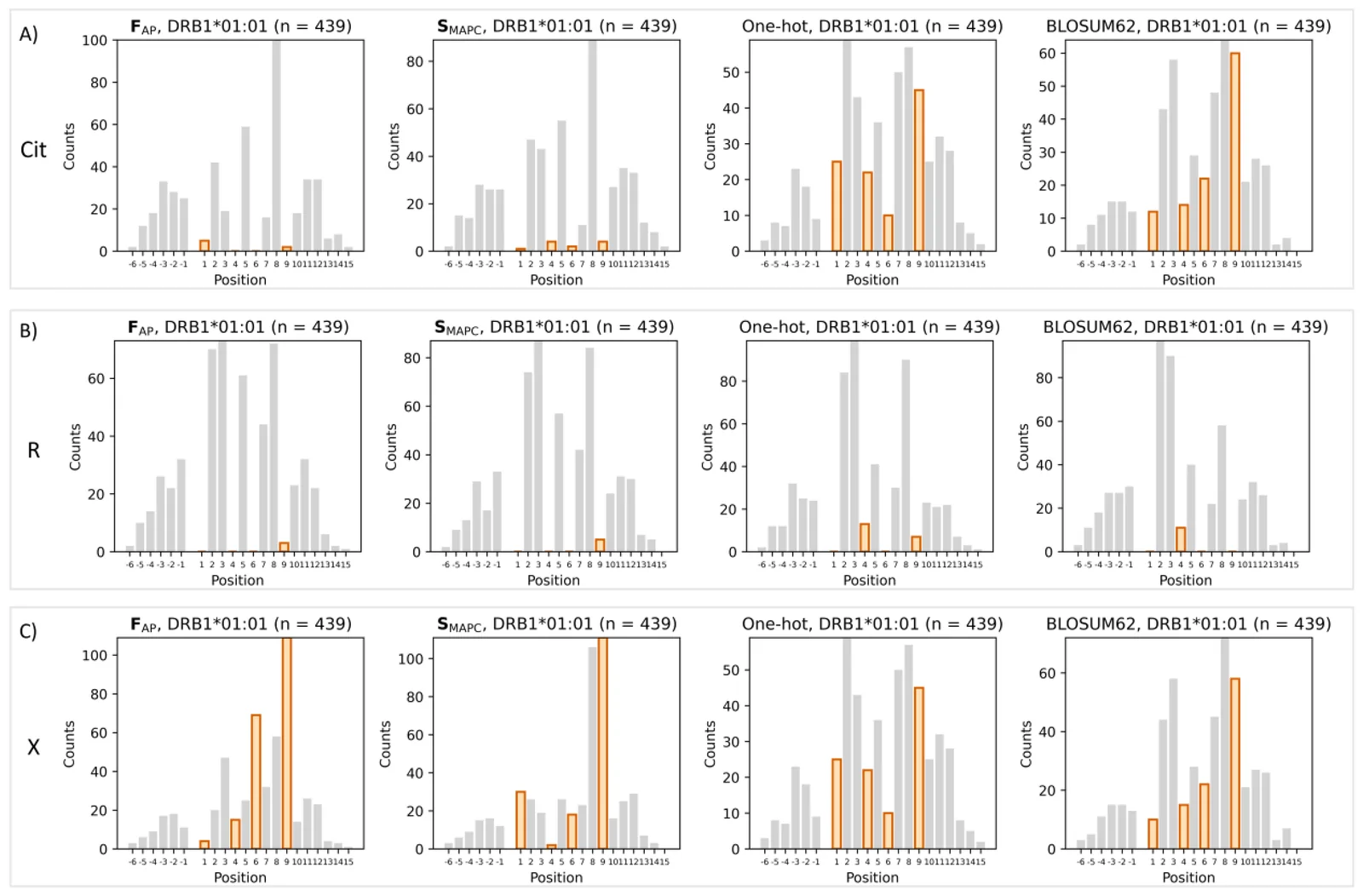
Citrulline positional frequency in predicted binders for DRB1*01:01 under different encoding strategies. Positional occurrence of citrulline in top 1% predicted binders (n = 439) from citrullinated peptides [Rebak et al. (25)], comparing predictions from models trained with citrulline encoded as: **(A)** citrulline (chemical fingerprint for **F**_AP_ and **S**_MAPC_ and zero-vector for one-hot and BLOSUM62), **(B)** arginine (natural analogue), or **(C)** ‘X’ (unknown residue). X-axis shows position relative to the 9-mer binding core: positions 1–9 indicate core positions, negative numbers indicate N-terminal flanking positions, and numbers >9 indicate C-terminal flanking positions. Orange bars highlight frequency at anchor positions (P1, P4, P6, P9); gray bars show frequency at all other positions. Results for DRB1*04:01 are reported in **Supplementary Figure 20**.

The positional distribution of citrulline in predicted binders provides a mechanistic explanation for the comparable performance observed between residue-level fingerprints and sequence-based encodings (**Figure 7**; **Supplementary Figure 20**). When citrulline is explicitly encoded or substituted with arginine, it rarely appears at the four canonical anchor positions (P1, P4, P6, P9). In contrast, when encoded as a zero vector, citrulline shows increased frequency at anchor positions, indicating the model’s inability to distinguish the modification from missing information. These results are in line with earlier findings (44) and suggest that binding affinity is mainly determined by NAAs at critical anchor positions, allowing sequence-based encodings to achieve performance comparable to residue-level fingerprints despite lacking explicit chemical information. The advantage of explicit chemical encoding may therefore depend on whether modifications occur at positions critical for binding.

### 3.4 Implementation

The developed models have been included into a prediction tool to facilitate pMHCII binding prediction on new peptides. MHC class II binding affinity for new peptide sequences can be predicted using this tool, which accepts both NAAs and NNAAs, with NNAAs encoded in square brackets (e.g., citrulline encoded as [Cit]). After specifying the molecular representation and allele, the tool outputs predicted binding scores with extracted binding cores for each peptide.

## 4 Discussion

We have presented a machine learning approach combining chemical fingerprints with sequence information to predict MHCII binding affinity for both NAA- and NNAA-based peptides. While chemical fingerprints have been widely used in small-molecule drug discovery, to our knowledge their application to peptide-MHCII binding prediction has not been previously explored. A key finding is that residue-level chemical fingerprints (direct-encoding and similarity-based) achieved performances comparable to sequence-based encodings (BLOSUM62 and one-hot) for predicting MHC class I and II binding with NAA-based peptides while accurately identifying binding cores and motifs. In contrast, peptide-level fingerprints demonstrated poor performance due to loss of positional information essential for binding prediction. For citrullinated peptides, residue-level fingerprints showed similar linear correlation performance compared to sequence-based encodings, with marginally improved quantitative prediction accuracy when citrullinated peptides were included during training. This is consistent with citrulline having a close natural analogue in arginine and rarely occupying anchor positions. In therapeutic peptide design, however, NNAAs without a direct natural analogue are increasingly incorporated, where sequence-based encodings cannot adequately represent the modification. This is a scenario where chemical encoding is expected to provide a clear advantage, and future evaluation should therefore focus on peptides carrying such modifications to fully assess this potential.

A key bottleneck in predicting binding affinity for NNAA-based peptides is the scarcity of experimental data. While the IEDB database contains valuable data for biologically relevant post-translational modifications, including phosphorylation and deamidation, we did not incorporate these modifications due to several limitations, including lack of or insufficient quantitative binding data, and ambiguous annotations. Moreover, these modifications represent only a small fraction of the chemical diversity employed in modern drug design. Medicinal chemists routinely incorporate diverse synthetic NNAAs into peptide therapeutics to enhance pharmacological properties. Unfortunately, experimental peptide-MHCII binding data for these modifications is largely unavailable from public data sets, limiting the evaluation of encoding strategies for peptides. Expanding the data set by including a broader range of modifications at various positions, particularly anchor residues, would improve prediction accuracy and further clarify the impact of chemical encoding on binding affinity. Physics-based computational methods have shown promise in predicting binding affinities for peptide-MHC interactions and may be considered in future work.

Two types of residue-level chemical fingerprints were investigated, showing comparable performance in predicting binding affinity for citrullinated peptides. However, different characteristics should be considered when selecting between these approaches. Direct-encoding fingerprints create a direct mapping from amino acid positions to their chemical features. In contrast, similarity-based fingerprints encode pairwise relationships between amino acids, requiring an additional computational step to calculate similarity coefficients for all amino acid pairs. The two representations differ in dimensionality: for 9-mer cores, direct-encoding fingerprints generate 9 × 256 = 2,304 features for most representations, while similarity-based fingerprints produce 9 × 22 = 198 features, scaling with the amino acid vocabulary size. For small data sets, the high feature-to-sample ratio of direct-encoding fingerprints may increase the risk of overfitting, potentially requiring regularization techniques or dimensionality reduction methods. Conversely, direct-encoding fingerprints offer greater flexibility for incorporating NNAAs not encountered during training, which can be directly encoded using their chemical fingerprints. While still possible for similarity-based fingerprints, this requires pre-allocating placeholder positions in the similarity matrix, which are populated with computed similarity scores when new residues are introduced.

Both approaches offer flexibility in accommodating different representations and similarity metrics, supporting future methodological improvements. In this work, we employed the Tanimoto similarity coefficient for similarity-based fingerprints, yielding a symmetric similarity measure analogous to BLOSUM62. Evaluation of alternative similarity metrics, such as the asymmetric Tversky index, could identify which best captures different chemical relationships relevant to pMHC binding prediction, for instance, the directional nature of amino acid substitutions. Beyond traditional chemical fingerprints, deep learning embeddings have been proposed for pMHC binding prediction (45), and could be integrated as alternative representations to assess their comparative performance for peptide-MHC class II binding prediction.

We tested residue-level fingerprints on single-allele pMHC prediction, with a particular focus on MHC class II molecules due to their central role in immunogenicity risk assessment and therapeutic protein development. However, MHC class II data sets are generally of lower quality compared to MHC class I data and characterized by greater experimental variability. Additionally, MHC class II binding predictions are typically more challenging due to the open-ended nature of the binding groove, which accommodates peptides of variable length and permits peptide flanking regions to influence binding. These factors complicate achieving good prediction performances for all alleles. Integrating pan-allele models that incorporate both binding affinity and elution data has demonstrated performance improvements for MHC class II predictions. The employed predictive model and representations are compatible with pan-allele frameworks, as they rely on the NNAlign (13) model architecture at the basis of the NetMHCIIpan methods (14), allowing integration without substantial architectural modifications. This extension would enable evaluation across a broader range of modifications for which elution data are available, such as phosphorylation (16).

By combining concepts of chemical fingerprints and sequence-based encodings, the proposed framework complements sequence-based encodings with explicit chemical encoding for pMHCII binding prediction. Citrullination provided a case study showing that the impact of chemical encoding may depend on whether modifications occur at anchor residues. While the full advantage of explicit chemical encoding remains to be demonstrated for NNAAs at positions critical for binding, our framework is designed to support this by encoding both NAAs and NNAAs, preserving positional information, and being compatible with pan-allele architectures. As experimental data sets for diverse NNAA-based peptides become available and with further validation, this approach could support immunogenicity risk assessment at the early discovery stage for emerging therapeutic modalities that incorporate NNAAs, such as therapeutic peptides, synthetic peptide vaccines and antibody drug conjugates.

## Data Availability

Publicly available datasets were analyzed in this study. This data can be found here: https://doi.org/10.5281/zenodo.19692117.
