# Supplementary material for: Beyond natural amino acids: extending immunogenicity risk assessment to modified peptides through chemical feature encoding. A case study on citrullination
Source: Front Immunol. 2026 Sep 9;17:1869623. doi: 10.3389/fimmu.2026.1869623 (PMC13597324; doi:10.3389/fimmu.2026.1869623)
Supplement: Supplementary file 1 [file DataSheet1.pdf]

Supplementary Material for: 'Beyond natural amino acids: Extending immunogenicity risk assessment to modified peptides through chemical feature encoding. A case study on citrullination'

Maria Cairoli[1], Morten Nielsen[2], Catherine Betts[3], Olga Obrezanova[4], Leonardo De Maria[1]

[1]Discovery Computation and AI, Hit Discovery, Discovery Sciences, Biopharmaceuticals R&D, AstraZeneca, Gothenburg, Sweden

[2]Department of Health Technology, Technical University of Denmark, Lyngby, Denmark

[3]Immune Safety, Clinical Pharmacology and Safety Sciences, Biopharmaceuticals R&D, AstraZeneca, Cambridge, UK

[4]Biologics Engineering, Oncology R&D, AstraZeneca, Cambridge, UK

Contents of this file:

- Supplementary Figures 1-20
- Supplementary Table 1

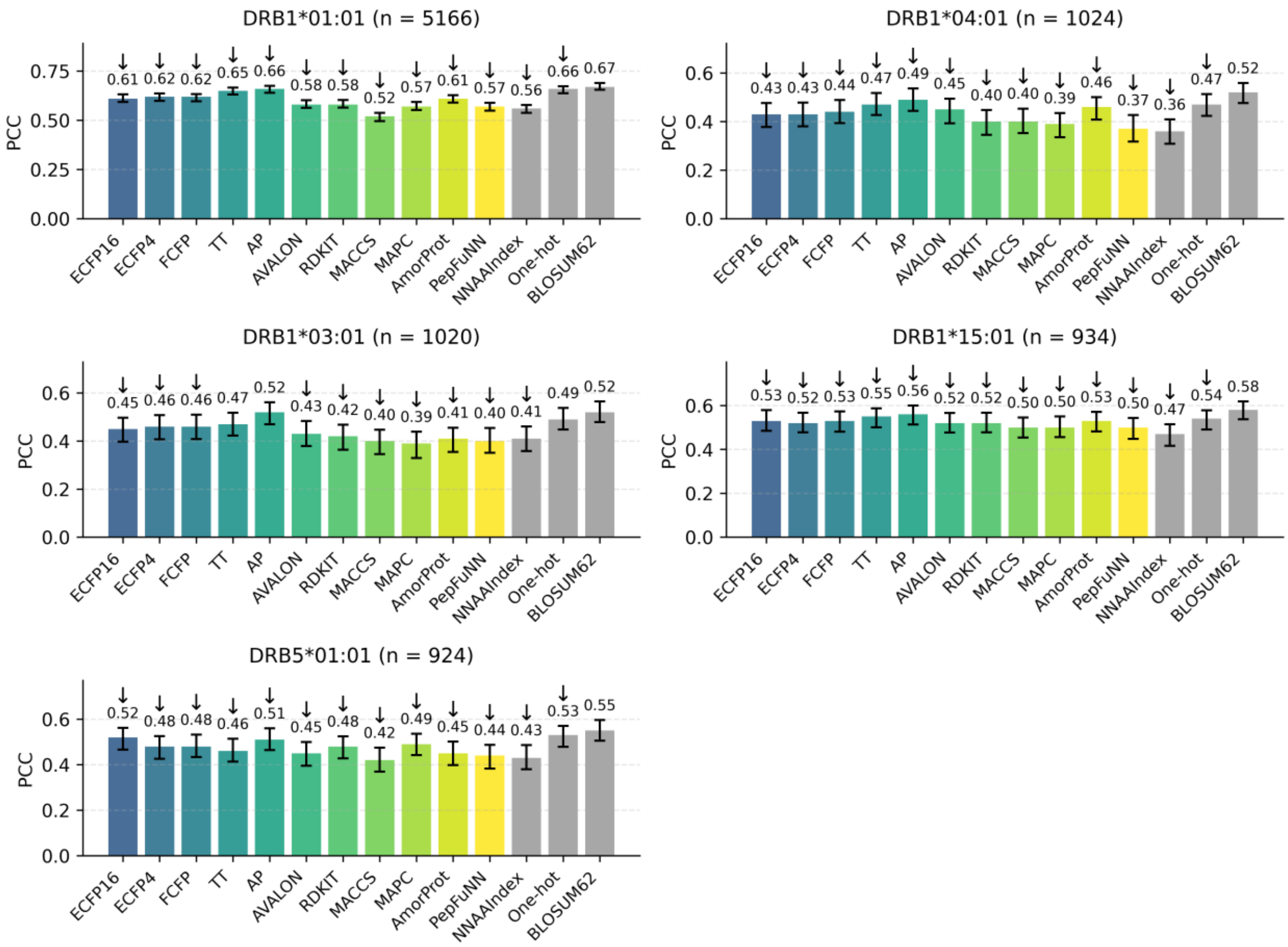

Supplementary Figure 1. PCC performance of peptide-level fingerprints for MHC class II binding prediction. PCC values from out-of-fold predictions for NAA-based peptides binding to selected HLA-DR alleles. Each subplot shows results for one allele with sample size indicated in the title. Gray bars represent sequence-based encodings; coloured bars represent peptide-level chemical fingerprints (see **Table 2** for details). Upward arrows indicate significantly better performance than BLOSUM62; downward arrows indicate significantly worse performance than BLOSUM62; absence of arrows indicates no significant difference from BLOSUM62 (paired Wilcoxon tests on per-peptide squared errors, FDR < 0.05, Benjamini-Hochberg correction for 13 pairwise comparisons per allele). Error bars report the 2.5^{th}-97.5^{th} percentiles of the bootstrap distribution (10,000 resamples).

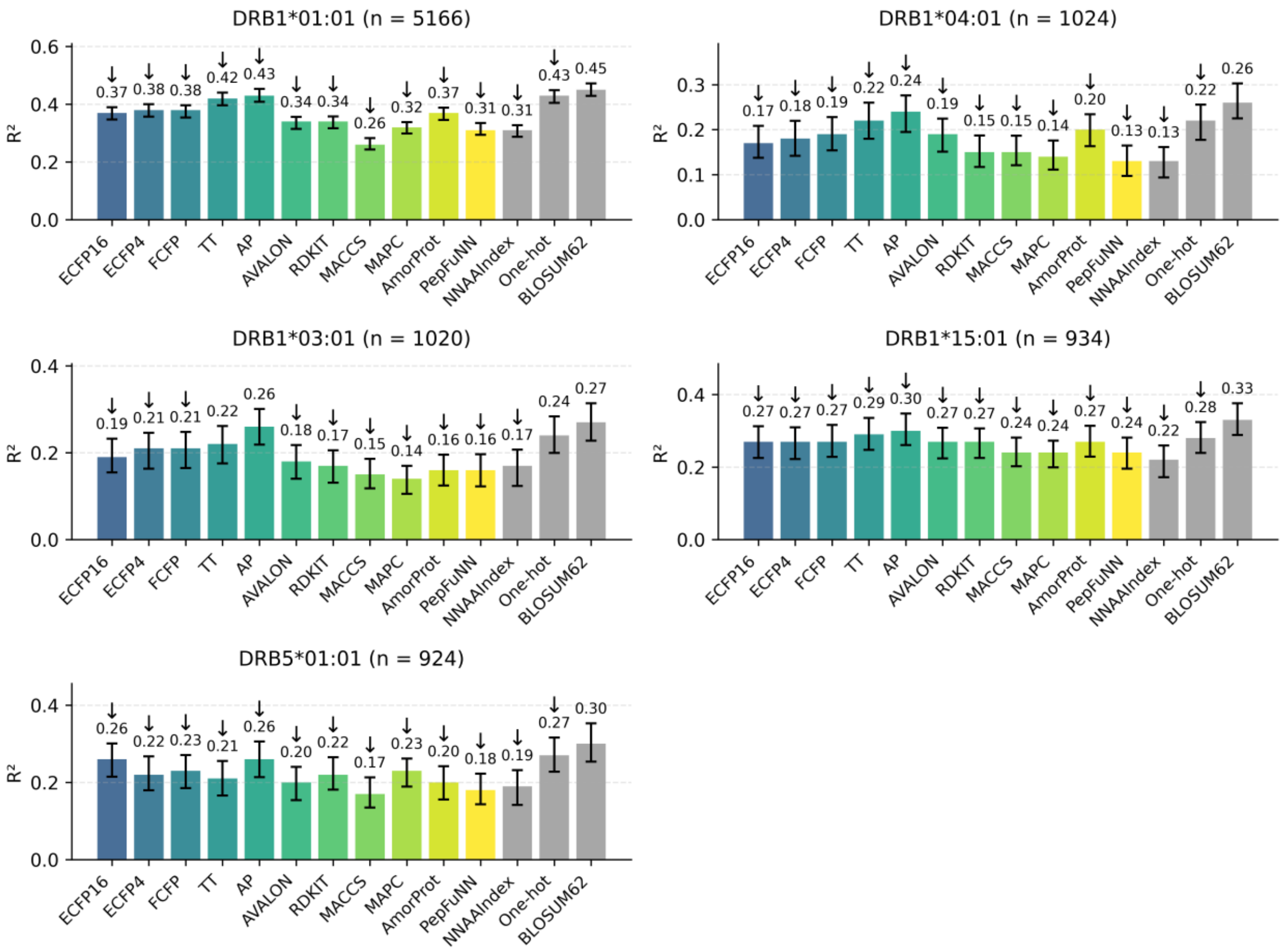

Supplementary Figure 2. R^2 performance of peptide-level fingerprints for MHC class II binding prediction. R^2 values from out-of-fold predictions for NAA-based peptides binding to selected HLA-DR alleles. Each subplot shows results for one allele with sample size indicated in the title. Gray bars represent sequence-based encodings; coloured bars represent peptide-level chemical fingerprints (see **Table 2** for details). Upward arrows indicate significantly better performance than BLOSUM62; downward arrows indicate significantly worse performance than BLOSUM62; absence of arrows indicates no significant difference from BLOSUM62 (paired Wilcoxon tests on per-peptide squared errors, FDR < 0.05, Benjamini-Hochberg correction for 13 pairwise comparisons per allele). Error bars report the 2.5^{th}-97.5^{th} percentiles of the bootstrap distribution (10,000 resamples).

Allele	Peptide	Core	ECFP16	ECFP4	FCFP	TT	AP	AVALON	RDKIT	MACCS	MAPC	AmorProt	PepFuNN	NNAAIndex	One-hot	BLOSUM62
DRB1*01:01	AAYSDQATPLLLSPR	YSDQATPLL	QATPLLLSP	QATPLLLSP	DQATPLLLS	QATPLLLSP	DQATPLLLS	QATPLLLSP	DQATPLLLS	QATPLLLSP	SDQATPLLL	QATPLLLSP	QATPLLLSP	YSDQATPLL	YSDQATPLL	YSDQATPLL
DRB1*01:01	AFVKQNAAALA	FVKQNAAAL	VKQNAAALA	VKQNAAALA	VKQNAAALA	VKQNAAALA	VKQNAAALA	VKQNAAALA	VKQNAAALA	VKQNAAALA	AFVKQNAAA	VKQNAAALA	VKQNAAALA	FVKQNAAAL	FVKQNAAAL	FVKQNAAAL
DRB1*01:01	AGFKGEQGPKGEPG	FKGEQGPKG	EQGPKGEPG	GEQGPKGEP	GEQGPKGEP	GEQGPKGEP	GEQGPKGEP	GEQGPKGEP	GEQGPKGEP	GEQGPKGEP	EQGPKGEPG	EQGPKGEPG	GEQGPKGEP	FKGEQGPKG	FKGEQGPKG	FKGEQGPKG
DRB1*01:01	GELIGILNAAKVPAD	IGILNAAKV	ILNAAKVPA	ILNAAKVPA	ILNAAKVPA	ILNAAKVPA	ILNAAKVPA	ILNAAKVPA	ILNAAKVPA	LNAAKVPAD	GELIGILNA	GILNAAKVP	GILNAAKVP	IGILNAAKV	IGILNAAKV	IGILNAAKV
DRB1*01:01	PEVIPMFSALSEGATP	VIPMFSALS	SALSEGATP	SALSEGATP	IPMFSALSE	SALSEGATP	SALSEGATP	IPMFSALSE	SALSEGATP	SALSEGATP	SALSEGATP	SALSEGATP	FSALSEGAT	VIPMFSALS	VIPMFSALS	VIPMFSALS
DRB1*01:01	PKYVKQNTLKLAT	YVKQNTLKL	KQNTLKLAT	KQNTLKLAT	KQNTLKLAT	KQNTLKLAT	VKQNTLKLA	VKQNTLKLA	VKQNTLKLA	KQNTLKLAT	KQNTLKLAT	KQNTLKLAT	KQNTLKLAT	YVKQNTLKL	YVKQNTLKL	YVKQNTLKL
DRB1*01:01	VGSDWRFLRGYHQYA	WRFLRGYHQ	FLRGYHQYA	FLRGYHQYA	FLRGYHQYA	RFLRGYHQY	FLRGYHQYA	RFLRGYHQY	FLRGYHQYA	RFLRGYHQY	FLRGYHQYA	FLRGYHQYA	FLRGYHQYA	WRFLRGYHQ	WRFLRGYHQ	WRFLRGYHQ
DRB1*03:01	PVSKMRMATPLLMQA	MRMATPLLM	VSKMRMATP	VSKMRMATP	VSKMRMATP	PVSKMRMAT	VSKMRMATP	VSKMRMATP	PVSKMRMAT	VSKMRMATP	PVSKMRMAT	MRMATPLLM	RMATPLLMQ	MRMATPLLM	MRMATPLLM	MRMATPLLM
DRB1*04:01	AYMRADAAAGGA	MRADAAAGG	RADAAAGGA	AYMRADAAA	RADAAAGGA	MRADAAAGG	AYMRADAAA	AYMRADAAA	MRADAAAGG	MRADAAAGG	RADAAAGGA	MRADAAAGG	AYMRADAAA	MRADAAAGG	YMRADAAAG	YMRADAAAG
DRB1*04:01	PKYVKQNTLKLAT	YVKQNTLKL	KQNTLKLAT	KQNTLKLAT	KQNTLKLAT	KQNTLKLAT	KQNTLKLAT	KYVKQNTLK	VKQNTLKLA	KQNTLKLAT	KQNTLKLAT	KQNTLKLAT	KQNTLKLAT	YVKQNTLKL	YVKQNTLKL	YVKQNTLKL
DRB1*15:01	ENPVVHFFKNIVTPR	VHFFKNIVT	VHFFKNIVT	VHFFKNIVT	VVHFFKNIV	VHFFKNIVT	VVHFFKNIV	VHFFKNIVT	VHFFKNIVT	VHFFKNIVT	FFKNIVTPR	HFFKNIVTP	VVHFFKNIV	VHFFKNIVT	VHFFKNIVT	VHFFKNIVT
DRB1*15:01	ENPVVHFFKNIVTPRGGSGGGGG	VHFFKNIVT	VVHFFKNIV	VHFFKNIVT	VVHFFKNIV	VHFFKNIVT	VVHFFKNIV	VHFFKNIVT	VHFFKNIVT	VVHFFKNIV	HFFKNIVTP	VVHFFKNIV	VVHFFKNIV	VHFFKNIVT	VHFFKNIVT	VHFFKNIVT
DRB5*01:01	GGVYHFVKKHVHES	YHFVKKHVH	FVKKHVHES	FVKKHVHES	FVKKHVHES	FVKKHVHES	HFVKKHVHE	HFVKKHVHE	HFVKKHVHE	FVKKHVHES	FVKKHVHES	FVKKHVHES	HFVKKHVHE	YHFVKKHVH	FVKKHVHES	YHFVKKHVH
DRB5*01:01	NPVVHFFKNIVTPRTPPPSQ	FKNIVTPRT	IVTPRTPPP	IVTPRTPPP	FKNIVTPRT	KNIVTPRTP	NIVTPRTPP	IVTPRTPPP	IVTPRTPPP	IVTPRTPPP	VHFFKNIVT	IVTPRTPPP	IVTPRTPPP	FKNIVTPRT	FKNIVTPRT	FKNIVTPRT
DRB5*01:01	VHFFKNIVTPRTPGG	FKNIVTPRT	VHFFKNIVT	VHFFKNIVT	NIVTPRTPG	KNIVTPRTP	NIVTPRTPG	IVTPRTPGG	NIVTPRTPG	NIVTPRTPG	IVTPRTPGG	IVTPRTPGG	VHFFKNIVT	FFKNIVTPR	FKNIVTPRT	FKNIVTPRT
**Matching**			7%	13%	7%	20%	0%	13%	20%	13%	0%	13%	0%	93%	87%	93%

Supplementary Figure 3. Binding core predictions from peptide-level fingerprints compared to experimental references. Predicted versus reference binding cores for 15 HLA-DR restricted peptides as determined from protein structures (Protein Data Bank, retrieved from Ref. (1)). Columns indicate HLA-DR restriction, peptide sequence, reference core (experimentally determined), and predicted cores for each peptide-level fingerprint. Green: matching predictions; red: divergent predictions.

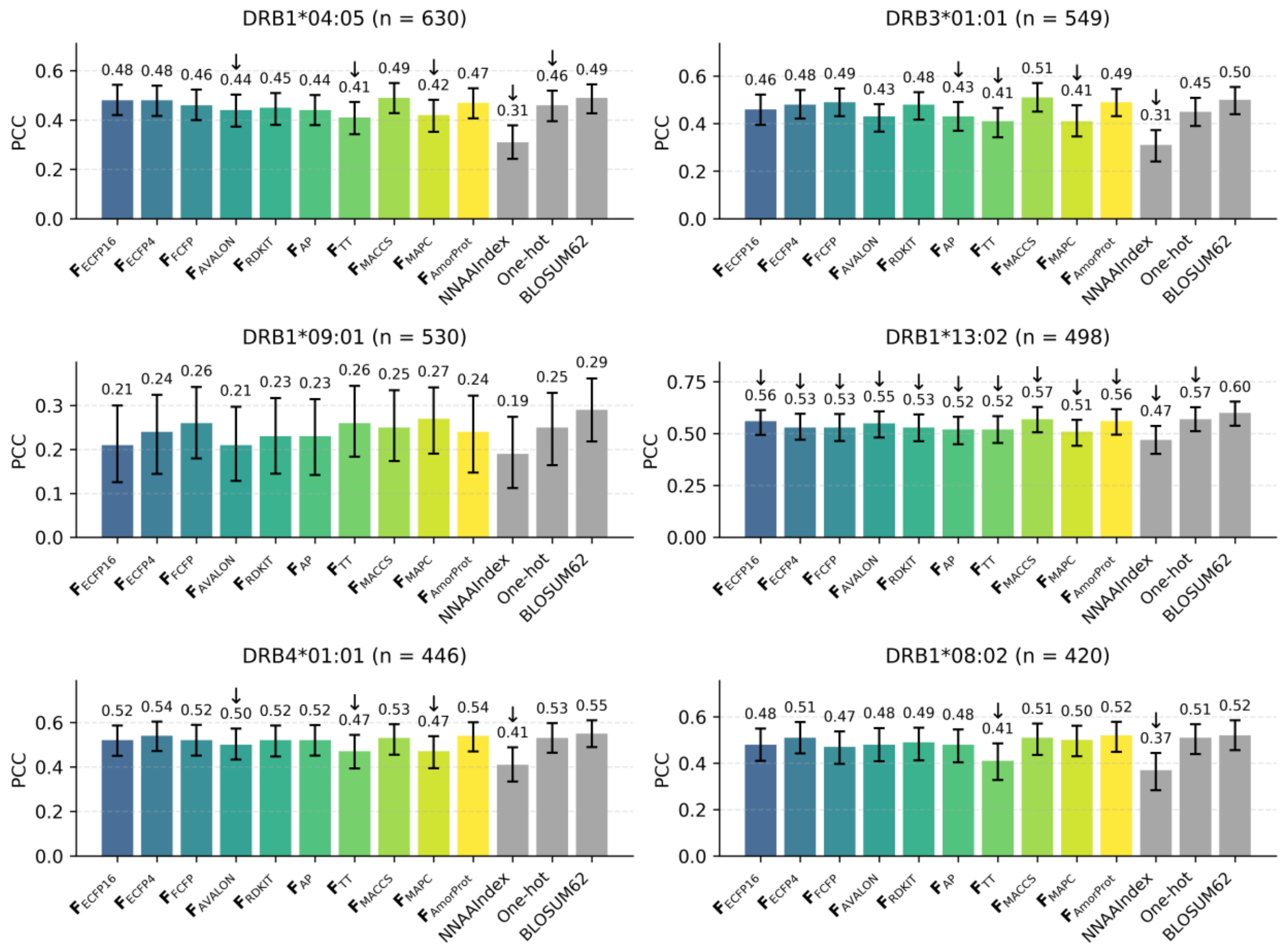

Supplementary Figure 4. PCC performance of direct-encoding fingerprints for MHC class II binding prediction. PCC values from out-of-fold predictions for NAA-based peptides binding to selected HLA-DR alleles. Each subplot shows results for one allele with sample size indicated in the title. Gray bars represent sequence-based encodings; coloured bars represent direct-encoding chemical fingerprints. Upward arrows indicate significantly better performance than BLOSUM62; downward arrows indicate significantly worse performance than BLOSUM62; absence of arrows indicates no significant difference from BLOSUM62 (paired Wilcoxon tests on per-peptide squared errors, FDR < 0.05, Benjamini-Hochberg correction for 12 pairwise comparisons per allele). Error bars report the 2.5^{th}-97.5^{th} percentiles of the bootstrap distribution (10,000 resamples).

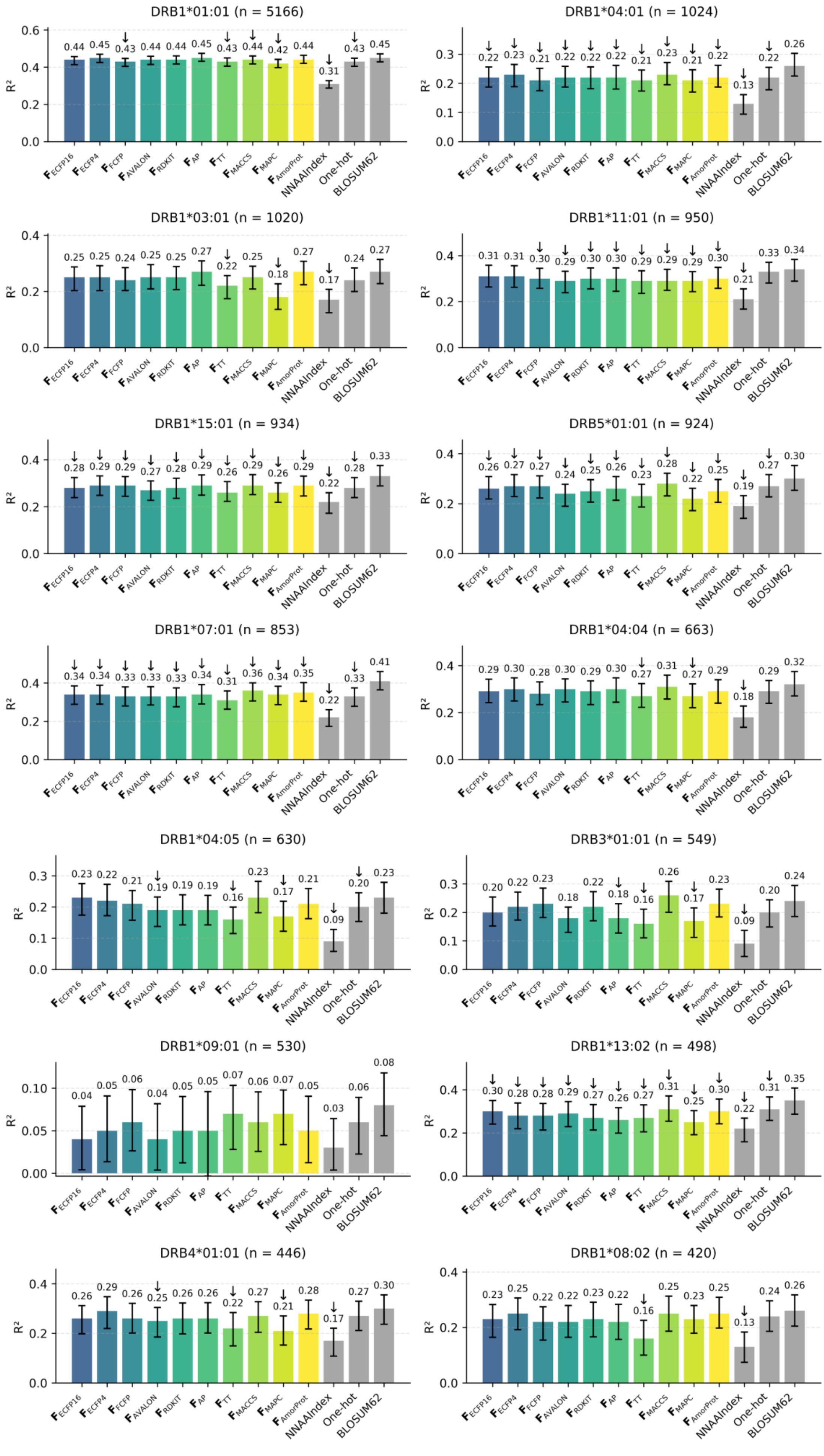
DRB1*01:01 (n = 5166)
0.44 0.45 0.43 0.44 0.44 0.45 0.43 0.44 0.42 0.44 0.31 0.43 0.45
DRB1*04:01 (n = 1024)
0.22 0.23 0.21 0.22 0.22 0.22 0.21 0.23 0.21 0.22 0.13 0.22 0.26
DRB1*03:01 (n = 1020)
0.25 0.25 0.24 0.25 0.25 0.27 0.22 0.25 0.18 0.27 0.17 0.24 0.27
DRB1*11:01 (n = 950)
0.31 0.31 0.30 0.29 0.30 0.30 0.29 0.29 0.29 0.30 0.21 0.33 0.34
DRB1*15:01 (n = 934)
0.28 0.29 0.29 0.27 0.28 0.29 0.26 0.29 0.26 0.29 0.22 0.28 0.33
DRB5*01:01 (n = 924)
0.26 0.27 0.27 0.24 0.25 0.26 0.23 0.28 0.22 0.25 0.19 0.27 0.30
DRB1*07:01 (n = 853)
0.34 0.34 0.33 0.33 0.33 0.34 0.31 0.36 0.34 0.35 0.22 0.33 0.41
DRB1*04:04 (n = 663)
0.29 0.30 0.28 0.30 0.29 0.30 0.27 0.31 0.27 0.29 0.18 0.29 0.32
DRB1*04:05 (n = 630)
0.23 0.22 0.21 0.19 0.19 0.19 0.16 0.23 0.17 0.21 0.09 0.20 0.23
DRB3*01:01 (n = 549)
0.20 0.22 0.23 0.18 0.22 0.18 0.16 0.26 0.17 0.23 0.09 0.20 0.24
DRB1*09:01 (n = 530)
0.04 0.05 0.06 0.04 0.05 0.05 0.07 0.06 0.07 0.05 0.03 0.06 0.08
DRB1*13:02 (n = 498)
0.30 0.28 0.28 0.29 0.27 0.26 0.27 0.31 0.25 0.30 0.22 0.31 0.35
DRB4*01:01 (n = 446)
0.26 0.29 0.26 0.25 0.26 0.26 0.22 0.27 0.21 0.28 0.17 0.27 0.30
DRB1*08:02 (n = 420)
0.23 0.25 0.22 0.22 0.23 0.22 0.16 0.25 0.23 0.25 0.13 0.24 0.26
R²
F_{ECFP16} F_{ECFP4} F_{FCFP} F_{AVALON} F_{RDKIT} F_{AP} F_{TT} F_{MACCS} F_{MAPC} $F_{AmorProt}$ NNAAIndex One-hot BLOSUM62

Supplementary Figure 5. R^2 performance of direct-encoding fingerprints for MHC class II binding prediction. R^2 values from out-of-fold predictions for NAA-based peptides binding to selected HLA-DR alleles. Each subplot shows results for one allele with sample size indicated in the title. Gray bars represent sequence-based encodings; coloured bars represent direct-encoding chemical fingerprints. Upward arrows indicate significantly better performance than BLOSUM62; downward arrows indicate significantly worse performance than BLOSUM62; absence of arrows indicates no significant difference from BLOSUM62 (paired Wilcoxon tests on per-peptide squared errors, FDR < 0.05, Benjamini-Hochberg correction for 12 pairwise comparisons per allele). Error bars report the 2.5th-97.5th percentiles of the bootstrap distribution (10,000 resamples).

Allele	Peptide	Core	F_{ECFP16}	F_{ECFP4}	F_{FCFP}	F_{AVALON}	F_{RDKIT}	F_{AP}	F_{TT}	F_{MACCS}	F_{MAPC}	$F_{AmorProt}$	NNAAIndex	One-hot	BLOSUM62
**DRB1*01:01**	AAYSDQATPLLLSPR	YSDQATPLL	YSDQATPLL	YSDQATPLL	YSDQATPLL	YSDQATPLL	YSDQATPLL	YSDQATPLL	YSDQATPLL	YSDQATPLL	YSDQATPLL	YSDQATPLL	YSDQATPLL	YSDQATPLL	YSDQATPLL
**DRB1*01:01**	AFVKQNAAALA	FVKQNAAAL	FVKQNAAAL	FVKQNAAAL	FVKQNAAAL	FVKQNAAAL	FVKQNAAAL	FVKQNAAAL	FVKQNAAAL	FVKQNAAAL	FVKQNAAAL	FVKQNAAAL	FVKQNAAAL	FVKQNAAAL	FVKQNAAAL
**DRB1*01:01**	AGFKGEQGPKGEPG	FKGEQGPKG	FKGEQGPKG	FKGEQGPKG	FKGEQGPKG	FKGEQGPKG	FKGEQGPKG	FKGEQGPKG	FKGEQGPKG	FKGEQGPKG	FKGEQGPKG	FKGEQGPKG	FKGEQGPKG	FKGEQGPKG	FKGEQGPKG
**DRB1*01:01**	GELIGILNAAKVPAD	IGILNAAKV	IGILNAAKV	IGILNAAKV	IGILNAAKV	IGILNAAKV	IGILNAAKV	IGILNAAKV	IGILNAAKV	IGILNAAKV	IGILNAAKV	IGILNAAKV	IGILNAAKV	IGILNAAKV	IGILNAAKV
**DRB1*01:01**	PEVIPMFSALSEGATP	VIPMFSALS	VIPMFSALS	VIPMFSALS	VIPMFSALS	VIPMFSALS	VIPMFSALS	VIPMFSALS	VIPMFSALS	VIPMFSALS	VIPMFSALS	VIPMFSALS	VIPMFSALS	VIPMFSALS	VIPMFSALS
**DRB1*01:01**	PKYVKQNTLKLAT	YVKQNTLKL	YVKQNTLKL	YVKQNTLKL	YVKQNTLKL	YVKQNTLKL	YVKQNTLKL	YVKQNTLKL	YVKQNTLKL	YVKQNTLKL	YVKQNTLKL	YVKQNTLKL	YVKQNTLKL	YVKQNTLKL	YVKQNTLKL
**DRB1*01:01**	VGSDWRFLRGYHQYA	WRFLRGYHQ	WRFLRGYHQ	WRFLRGYHQ	WRFLRGYHQ	WRFLRGYHQ	WRFLRGYHQ	WRFLRGYHQ	WRFLRGYHQ	WRFLRGYHQ	WRFLRGYHQ	WRFLRGYHQ	WRFLRGYHQ	WRFLRGYHQ	WRFLRGYHQ
**DRB1*03:01**	PVSKMRMATPLLMQA	MRMATPLLM	MRMATPLLM	MRMATPLLM	MRMATPLLM	MRMATPLLM	MRMATPLLM	MRMATPLLM	MRMATPLLM	MRMATPLLM	MRMATPLLM	MRMATPLLM	MRMATPLLM	MRMATPLLM	MRMATPLLM
**DRB1*04:01**	AYMRADAAAGGA	MRADAAAGG	MRADAAAGG	MRADAAAGG	YMRADAAAG	YMRADAAAG	YMRADAAAG	MRADAAAGG	MRADAAAGG	MRADAAAGG	YMRADAAAG	MRADAAAGG	MRADAAAGG	YMRADAAAG	YMRADAAAG
**DRB1*04:01**	PKYVKQNTLKLAT	YVKQNTLKL	YVKQNTLKL	YVKQNTLKL	YVKQNTLKL	YVKQNTLKL	YVKQNTLKL	YVKQNTLKL	YVKQNTLKL	YVKQNTLKL	YVKQNTLKL	YVKQNTLKL	YVKQNTLKL	YVKQNTLKL	YVKQNTLKL
**DRB1*15:01**	ENPVVHFFKNIVTPR	VHFFKNIVT	VHFFKNIVT	VHFFKNIVT	VHFFKNIVT	VHFFKNIVT	VHFFKNIVT	VHFFKNIVT	VHFFKNIVT	VHFFKNIVT	VHFFKNIVT	VHFFKNIVT	VHFFKNIVT	VHFFKNIVT	VHFFKNIVT
**DRB1*15:01**	ENPVVHFFKNIVTPRGGSGGGGG	VHFFKNIVT	VHFFKNIVT	VHFFKNIVT	VHFFKNIVT	VHFFKNIVT	VHFFKNIVT	VHFFKNIVT	VHFFKNIVT	VHFFKNIVT	VHFFKNIVT	VHFFKNIVT	VHFFKNIVT	VHFFKNIVT	VHFFKNIVT
**DRB5*01:01**	GGVYHFVKKHVHES	YHFVKKHVH	FVKKHVHES	YHFVKKHVH	YHFVKKHVH	YHFVKKHVH	YHFVKKHVH	YHFVKKHVH	FVKKHVHES	YHFVKKHVH	YHFVKKHVH	YHFVKKHVH	YHFVKKHVH	FVKKHVHES	YHFVKKHVH
**DRB5*01:01**	NPVVHFFKNIVTPRTPPPSQ	FKNIVTPRT	FKNIVTPRT	FKNIVTPRT	FKNIVTPRT	FKNIVTPRT	FKNIVTPRT	FKNIVTPRT	FKNIVTPRT	FKNIVTPRT	FFKNIVTPR	FKNIVTPRT	FKNIVTPRT	FKNIVTPRT	FKNIVTPRT
**DRB5*01:01**	VHFFKNIVTPRTPGG	FKNIVTPRT	FKNIVTPRT	FKNIVTPRT	FKNIVTPRT	FKNIVTPRT	FKNIVTPRT	FKNIVTPRT	FKNIVTPRT	FKNIVTPRT	FFKNIVTPR	FKNIVTPRT	FFKNIVTPR	FKNIVTPRT	FKNIVTPRT
**Matching**			**93%**	**100%**	**93%**	**93%**	**93%**	**100%**	**93%**	**100%**	**80%**	**100%**	**93%**	**87%**	**93%**

Supplementary Figure 6. Binding core predictions from direct-encoding fingerprints compared to experimental references. Predicted versus reference binding cores for 15 HLA-DR restricted peptides as determined from protein structures (Protein Data Bank, retrieved from Ref. (1)). Columns indicate HLA-DR restriction, peptide sequence, reference core (experimentally determined), and predicted cores for each peptide-level fingerprint. Green: matching predictions; red: divergent predictions.

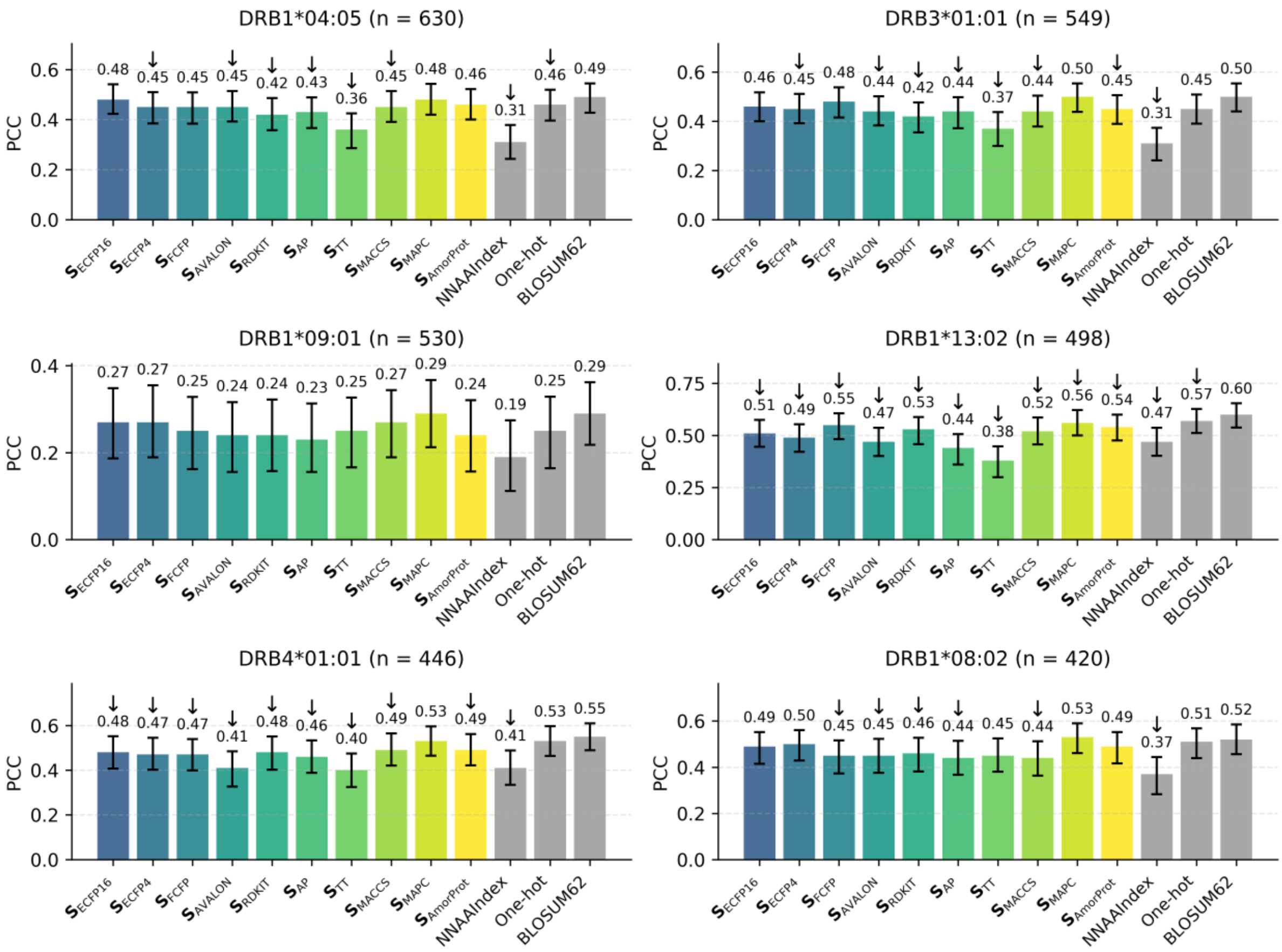

Supplementary Figure 7. PCC performance of similarity-based fingerprints for MHC class II binding prediction. PCC values from out-of-fold predictions for NAA-based peptides binding to selected HLA-DR alleles. Each subplot shows results for one allele with sample size indicated in the title. Gray bars represent sequence-based encodings; coloured bars represent similarity-based fingerprints. Upward arrows indicate significantly better performance than BLOSUM62; downward arrows indicate significantly worse performance than BLOSUM62; absence of arrows indicates no significant difference from BLOSUM62 (paired Wilcoxon tests on per-peptide squared errors, FDR < 0.05, Benjamini-Hochberg correction for 12 pairwise comparisons per allele). Error bars report the 2.5th-97.5th percentiles of the bootstrap distribution (10,000 resamples).

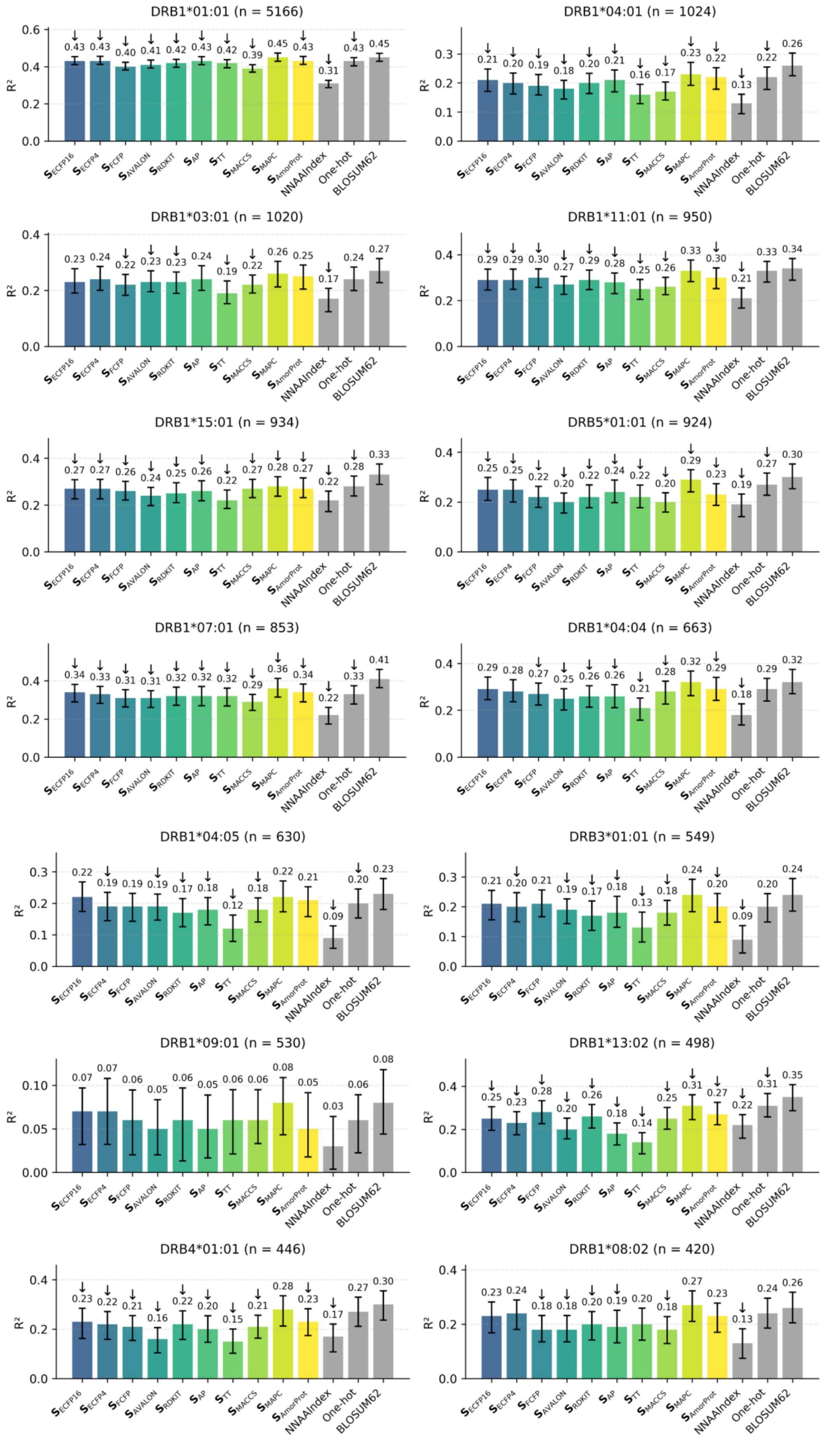
DRB1*01:01 (n = 5166)
0.43 0.43 0.40 0.41 0.42 0.43 0.42 0.39 0.45 0.43 0.31 0.43 0.45
DRB1*04:01 (n = 1024)
0.21 0.20 0.19 0.18 0.20 0.21 0.16 0.17 0.23 0.22 0.13 0.22 0.26
DRB1*03:01 (n = 1020)
0.23 0.24 0.22 0.23 0.23 0.24 0.19 0.22 0.26 0.25 0.17 0.24 0.27
DRB1*11:01 (n = 950)
0.29 0.29 0.30 0.27 0.29 0.28 0.25 0.26 0.33 0.30 0.21 0.33 0.34
DRB1*15:01 (n = 934)
0.27 0.27 0.26 0.24 0.25 0.26 0.22 0.27 0.28 0.27 0.22 0.28 0.33
DRB5*01:01 (n = 924)
0.25 0.25 0.22 0.20 0.22 0.24 0.22 0.20 0.29 0.23 0.19 0.27 0.30
DRB1*07:01 (n = 853)
0.34 0.33 0.31 0.31 0.32 0.32 0.32 0.29 0.36 0.34 0.22 0.33 0.41
DRB1*04:04 (n = 663)
0.29 0.28 0.27 0.25 0.26 0.26 0.21 0.28 0.32 0.29 0.18 0.29 0.32
DRB1*04:05 (n = 630)
0.22 0.19 0.19 0.19 0.17 0.18 0.12 0.18 0.22 0.21 0.09 0.20 0.23
DRB3*01:01 (n = 549)
0.21 0.20 0.21 0.19 0.17 0.18 0.13 0.18 0.24 0.20 0.09 0.20 0.24
DRB1*09:01 (n = 530)
0.07 0.07 0.06 0.05 0.06 0.05 0.06 0.06 0.08 0.05 0.03 0.06 0.08
DRB1*13:02 (n = 498)
0.25 0.23 0.28 0.20 0.26 0.18 0.14 0.25 0.31 0.27 0.22 0.31 0.35
DRB4*01:01 (n = 446)
0.23 0.22 0.21 0.16 0.22 0.20 0.15 0.21 0.28 0.23 0.17 0.27 0.30
DRB1*08:02 (n = 420)
0.23 0.24 0.18 0.18 0.20 0.19 0.20 0.18 0.27 0.23 0.13 0.24 0.26
R²
S_{ECFP16}
S_{ECFP4}
S_{FCFP}
S_{AVALON}
S_{RDKIT}
S_{AP}
S_{TT}
S_{MACCS}
S_{MAPC}
$S_{AmorProt}$
NNAAIndex
One-hot
BLOSUM62

Supplementary Figure 8. R^2 performance of similarity-based fingerprints for MHC class II binding prediction. R^2 values from out-of-fold predictions for NAA-based peptides binding to selected HLA-DR alleles. Each subplot shows results for one allele with sample size indicated in the title. Gray bars represent sequence-based encodings; coloured bars represent similarity-based fingerprints. Upward arrows indicate significantly better performance than BLOSUM62; downward arrows indicate significantly worse performance than BLOSUM62; absence of arrows indicates no significant difference from BLOSUM62 (paired Wilcoxon tests on per-peptide squared errors, FDR < 0.05, Benjamini-Hochberg correction for 12 pairwise comparisons per allele). Error bars report the 2.5th-97.5th percentiles of the bootstrap distribution (10,000 resamples).

Allele	Peptide	Core	S_{ECFP16}	S_{ECFP4}	S_{FCFP}	S_{AVALON}	S_{RDKIT}	S_{AP}	S_{TT}	S_{MACCS}	S_{MAPC}	$S_{AmorProt}$	NNAAIndex	One-hot	BLOSUM62
**DRB1*01:01**	AAYSDQATPLLLSPR	YSDQATPLL	YSDQATPLL	YSDQATPLL	YSDQATPLL	YSDQATPLL	YSDQATPLL	YSDQATPLL	YSDQATPLL	YSDQATPLL	YSDQATPLL	YSDQATPLL	YSDQATPLL	YSDQATPLL	YSDQATPLL
**DRB1*01:01**	AFVKQNAAALA	FVKQNAAAL	FVKQNAAAL	FVKQNAAAL	FVKQNAAAL	FVKQNAAAL	FVKQNAAAL	FVKQNAAAL	FVKQNAAAL	FVKQNAAAL	FVKQNAAAL	FVKQNAAAL	FVKQNAAAL	FVKQNAAAL	FVKQNAAAL
**DRB1*01:01**	AGFKGEQGPKGEPG	FKGEQGPKG	FKGEQGPKG	FKGEQGPKG	FKGEQGPKG	FKGEQGPKG	FKGEQGPKG	FKGEQGPKG	FKGEQGPKG	FKGEQGPKG	FKGEQGPKG	FKGEQGPKG	FKGEQGPKG	FKGEQGPKG	FKGEQGPKG
**DRB1*01:01**	GELIGILNAAKVPAD	IGILNAAKV	IGILNAAKV	IGILNAAKV	IGILNAAKV	IGILNAAKV	IGILNAAKV	IGILNAAKV	IGILNAAKV	IGILNAAKV	IGILNAAKV	IGILNAAKV	IGILNAAKV	IGILNAAKV	IGILNAAKV
**DRB1*01:01**	PEVIPMFSALSEGATP	VIPMFSALS	VIPMFSALS	VIPMFSALS	VIPMFSALS	VIPMFSALS	VIPMFSALS	VIPMFSALS	VIPMFSALS	VIPMFSALS	VIPMFSALS	VIPMFSALS	VIPMFSALS	VIPMFSALS	VIPMFSALS
**DRB1*01:01**	PKYVKQNTLKLAT	YVKQNTLKL	YVKQNTLKL	YVKQNTLKL	YVKQNTLKL	YVKQNTLKL	YVKQNTLKL	YVKQNTLKL	YVKQNTLKL	YVKQNTLKL	YVKQNTLKL	YVKQNTLKL	YVKQNTLKL	YVKQNTLKL	YVKQNTLKL
**DRB1*01:01**	VGSDWRFLRGYHQYA	WRFLRGYHQ	WRFLRGYHQ	WRFLRGYHQ	WRFLRGYHQ	WRFLRGYHQ	WRFLRGYHQ	WRFLRGYHQ	WRFLRGYHQ	WRFLRGYHQ	WRFLRGYHQ	WRFLRGYHQ	WRFLRGYHQ	WRFLRGYHQ	WRFLRGYHQ
**DRB1*03:01**	PVSKMRMATPLLMQA	MRMATPLLM	MRMATPLLM	MRMATPLLM	MRMATPLLM	MRMATPLLM	MRMATPLLM	MRMATPLLM	MRMATPLLM	MRMATPLLM	MRMATPLLM	MRMATPLLM	MRMATPLLM	MRMATPLLM	MRMATPLLM
**DRB1*04:01**	AYMRADAAAGGA	MRADAAAGG	YMRADAAAG	YMRADAAAG	YMRADAAAG	YMRADAAAG	YMRADAAAG	YMRADAAAG	YMRADAAAG	YMRADAAAG	YMRADAAAG	YMRADAAAG	MRADAAAGG	YMRADAAAG	YMRADAAAG
**DRB1*04:01**	PKYVKQNTLKLAT	YVKQNTLKL	YVKQNTLKL	YVKQNTLKL	YVKQNTLKL	YVKQNTLKL	YVKQNTLKL	YVKQNTLKL	YVKQNTLKL	YVKQNTLKL	YVKQNTLKL	YVKQNTLKL	YVKQNTLKL	YVKQNTLKL	YVKQNTLKL
**DRB1*15:01**	ENPVVHFFKNIVTPR	VHFFKNIVT	FFKNIVTPR	FFKNIVTPR	VHFFKNIVT	VHFFKNIVT	VHFFKNIVT	VHFFKNIVT	VHFFKNIVT	VHFFKNIVT	VHFFKNIVT	VHFFKNIVT	VHFFKNIVT	VHFFKNIVT	VHFFKNIVT
**DRB1*15:01**	ENPVVHFFKNIVTPRGGSGGGGG	VHFFKNIVT	VHFFKNIVT	VHFFKNIVT	VHFFKNIVT	VHFFKNIVT	VHFFKNIVT	VHFFKNIVT	VHFFKNIVT	VHFFKNIVT	VHFFKNIVT	VHFFKNIVT	VHFFKNIVT	VHFFKNIVT	VHFFKNIVT
**DRB5*01:01**	GGVYHFVKKHVHES	YHFVKKHVH	YHFVKKHVH	YHFVKKHVH	YHFVKKHVH	YHFVKKHVH	YHFVKKHVH	YHFVKKHVH	YHFVKKHVH	YHFVKKHVH	YHFVKKHVH	YHFVKKHVH	YHFVKKHVH	FVKKHVHES	YHFVKKHVH
**DRB5*01:01**	NPVVHFFKNIVTPRTPPPSQ	FKNIVTPRT	FKNIVTPRT	FKNIVTPRT	FKNIVTPRT	FKNIVTPRT	FKNIVTPRT	FKNIVTPRT	FKNIVTPRT	FKNIVTPRT	FKNIVTPRT	FKNIVTPRT	FKNIVTPRT	FKNIVTPRT	FKNIVTPRT
**DRB5*01:01**	VHFFKNIVTPRTPGG	FKNIVTPRT	FKNIVTPRT	FKNIVTPRT	FKNIVTPRT	FKNIVTPRT	FKNIVTPRT	FKNIVTPRT	FKNIVTPRT	FKNIVTPRT	FKNIVTPRT	FKNIVTPRT	FFKNIVTPR	FKNIVTPRT	FKNIVTPRT
**Matching**			87%	87%	93%	93%	93%	93%	93%	93%	93%	93%	93%	87%	93%

Supplementary Figure 9. Binding core predictions from similarity-based fingerprints compared to experimental references. Predicted versus reference binding cores for 15 HLA-DR restricted peptides as determined from protein structures (Protein Data Bank, retrieved from Ref. (1)). Columns indicate HLA-DR restriction, peptide sequence, reference core (experimentally determined), and predicted cores for each peptide-level fingerprint. Green: matching predictions; red: divergent predictions.

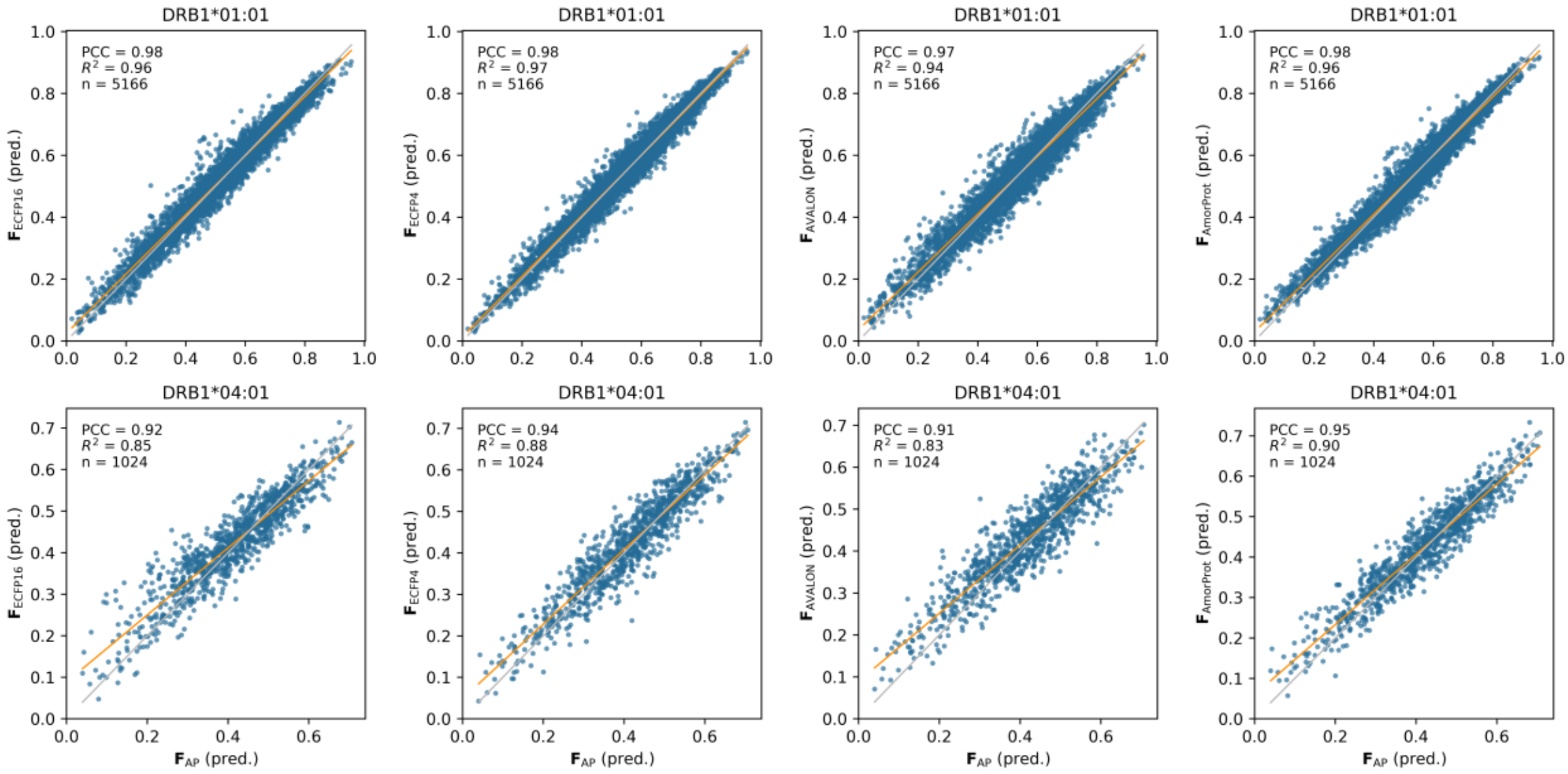

Supplementary Figure 10. Correlation among ba$_{score}$ predictions of F_{AP} against selected direct-encoding fingerprints for DRB1*01:01 and DRB1*04:01. Blue dots: NAA-based peptides; yellow line: fitted regression; gray line: identity line. Each panel shows results with PCC and R^2 performance metrics. n indicates the number of predicted samples.

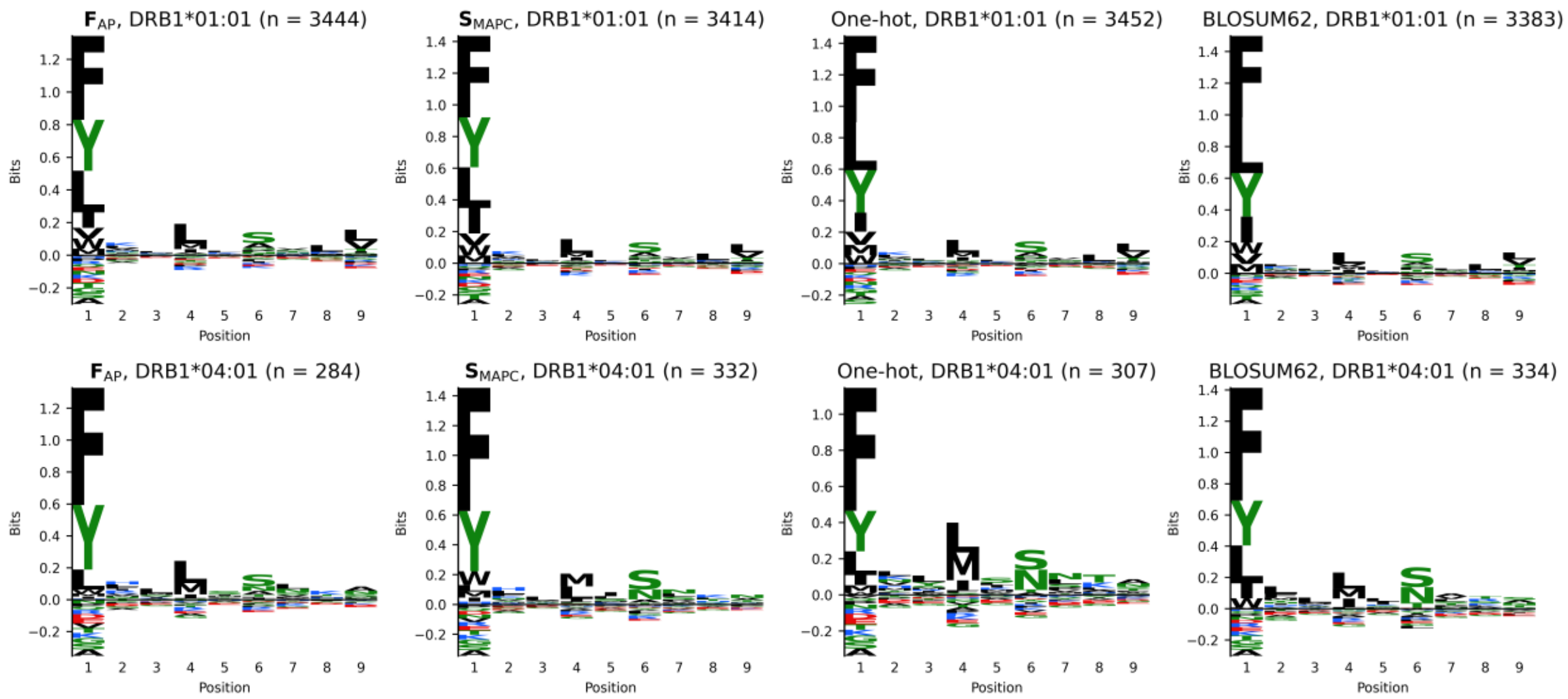

Supplementary Figure 11. Predicted binding motifs for MHC class II alleles. Sequence logos generated from out-of-fold predictions for peptides predicted as binders (ba$_{score}$ ≥ 0.5), considering $\mathbf{F}_{AP}$, $\mathbf{S}_{MAPC}$, one-hot and BLOSUM62 encodings. Logos display amino acid frequencies at each position, with letter height proportional to information content.

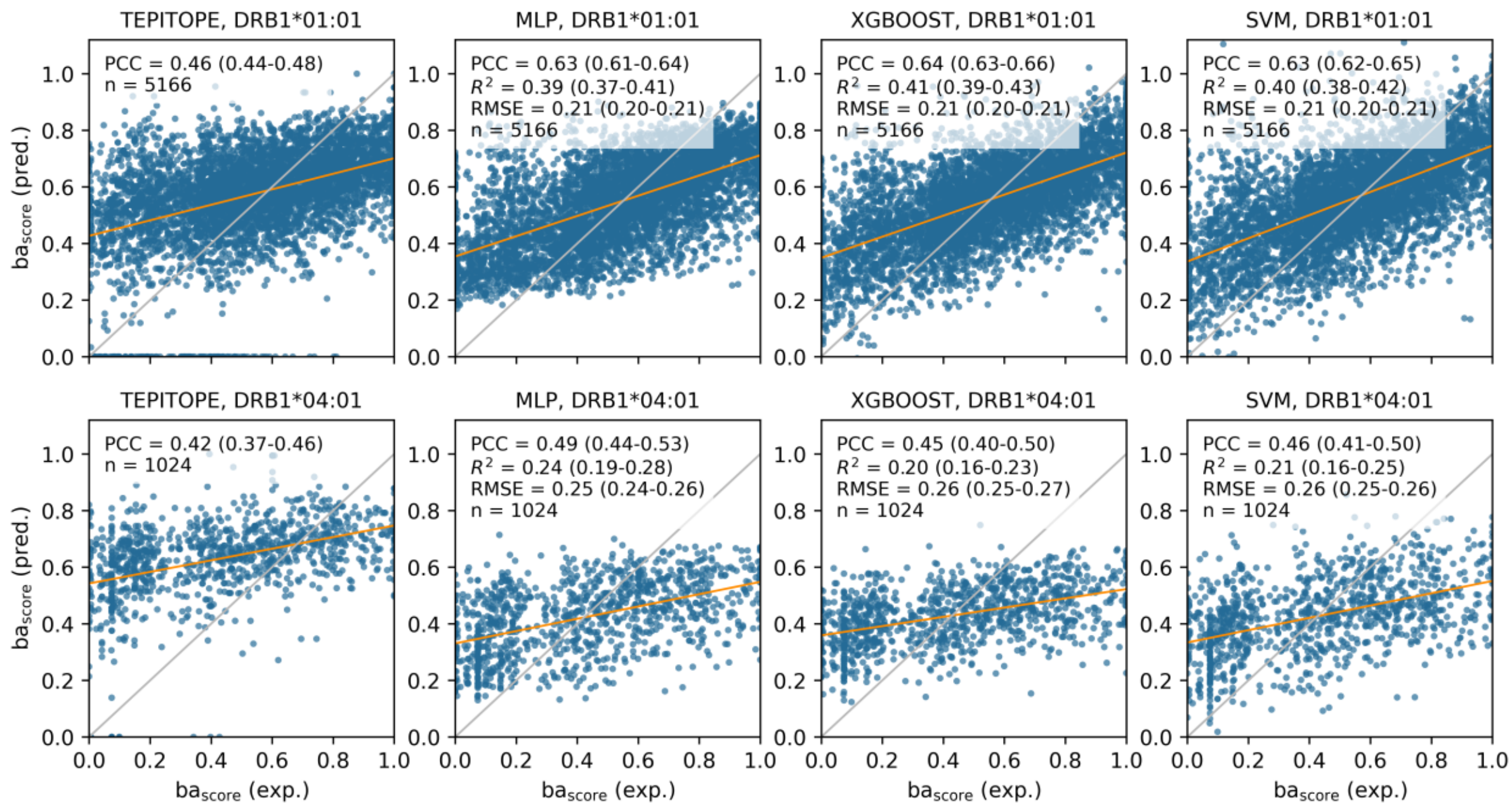

Supplementary Figure 12. Prediction performances for TEPITOPE-derived methods. Out-of-fold predictions for DRB1*01:01 and DRB1*04:01 using TEPITOPE-based methods. Blue dots represent NAA-based peptides. Yellow line: fitted regression; gray line: identity line. Raw TEPITOPE predictions were min-max scaled to [0,1] range for visual comparability with ba_{score} values. Each panel shows results for the different methods with PCC, R² and RMSE performance metrics, including the 2.5th and 97.5th percentiles of the bootstrap distribution (10,000 resamples). For raw TEPITOPE predictions, R² values are not reported due to scale differences between TEPITOPE scores and ba_{score} measurements. Since TEPITOPE scores are not calibrated to binding affinity values, this comparison should be interpreted as a reference baseline rather than a direct performance benchmark. MLP, XGBoost and SVM models were trained in cross-validation, following the same methodological strategy described in the manuscript. Hyperparameters were optimized using Optuna, as reported in **Supplementary Table S1**.

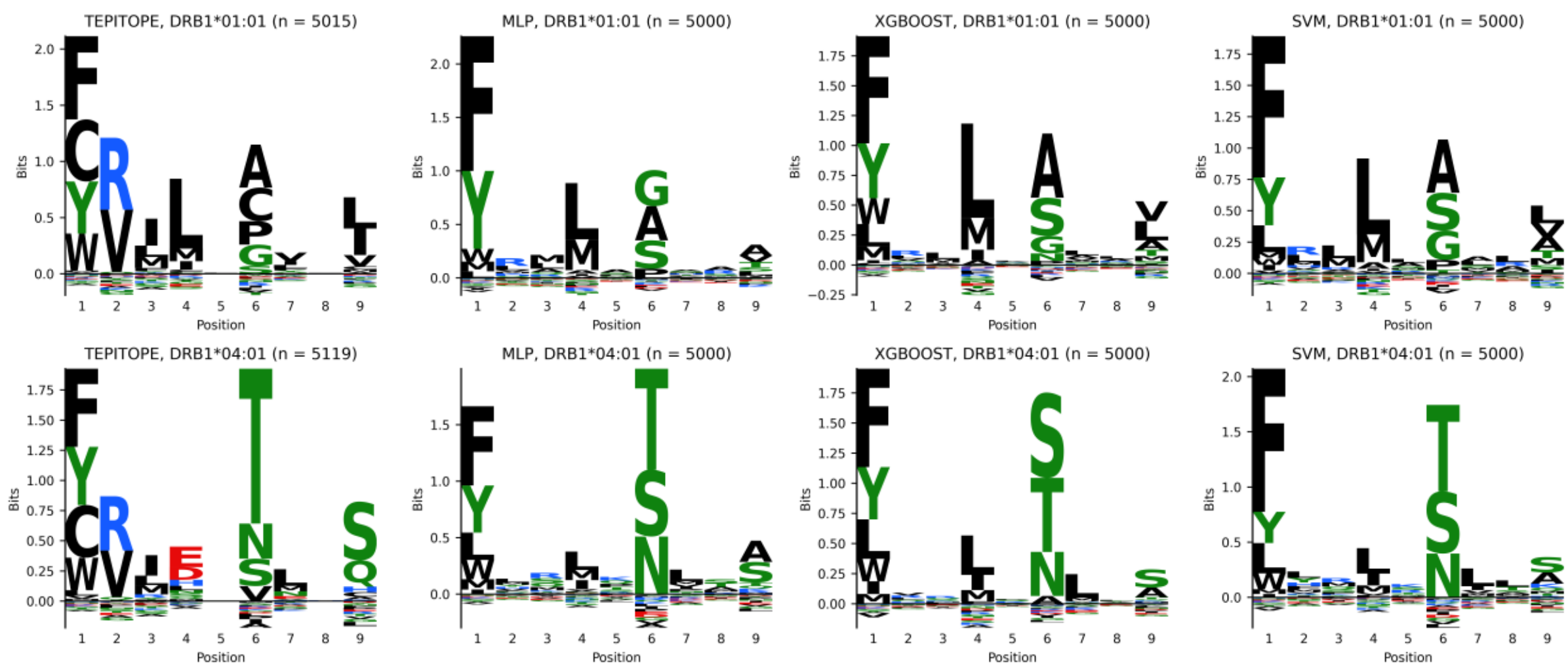

Supplementary Figure 13. Predicted binding motifs for TEPITOPE-derived methods. Sequence logos generated from the top 1% predicted binders among randomly sampled peptides from the UniProt human proteome for selected MHC class II alleles. Logos display amino acid frequencies at each position, with letter height proportional to information content.

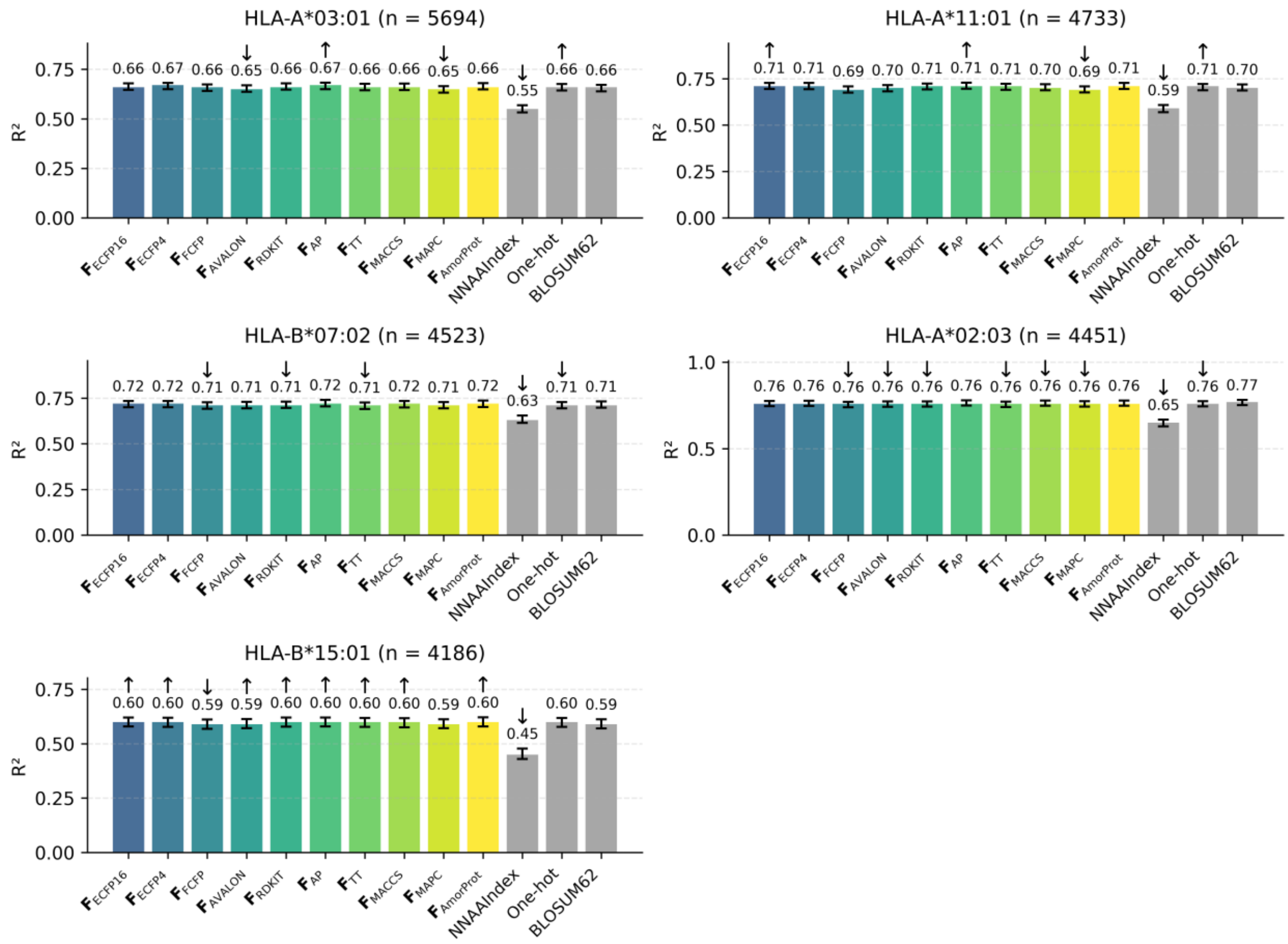

Supplementary Figure 14. R^2 performance of direct-encoding fingerprints for MHC class I binding prediction. R^2 values from out-of-fold predictions for NAA-based peptides binding to selected HLA-A and HLA-B alleles. Each subplot shows results for one allele with sample size indicated in the title. Gray bars represent sequence-based encodings; coloured bars represent direct-encoding fingerprints. Upward arrows indicate significantly better performance than BLOSUM62; downward arrows indicate significantly worse performance than BLOSUM62; absence of arrows indicates no significant difference from BLOSUM62 (paired Wilcoxon tests on per-peptide squared errors, FDR < 0.05, Benjamini-Hochberg correction for 12 pairwise comparisons per allele). Error bars report the 2.5th-97.5th percentiles of the bootstrap distribution (10,000 resamples).

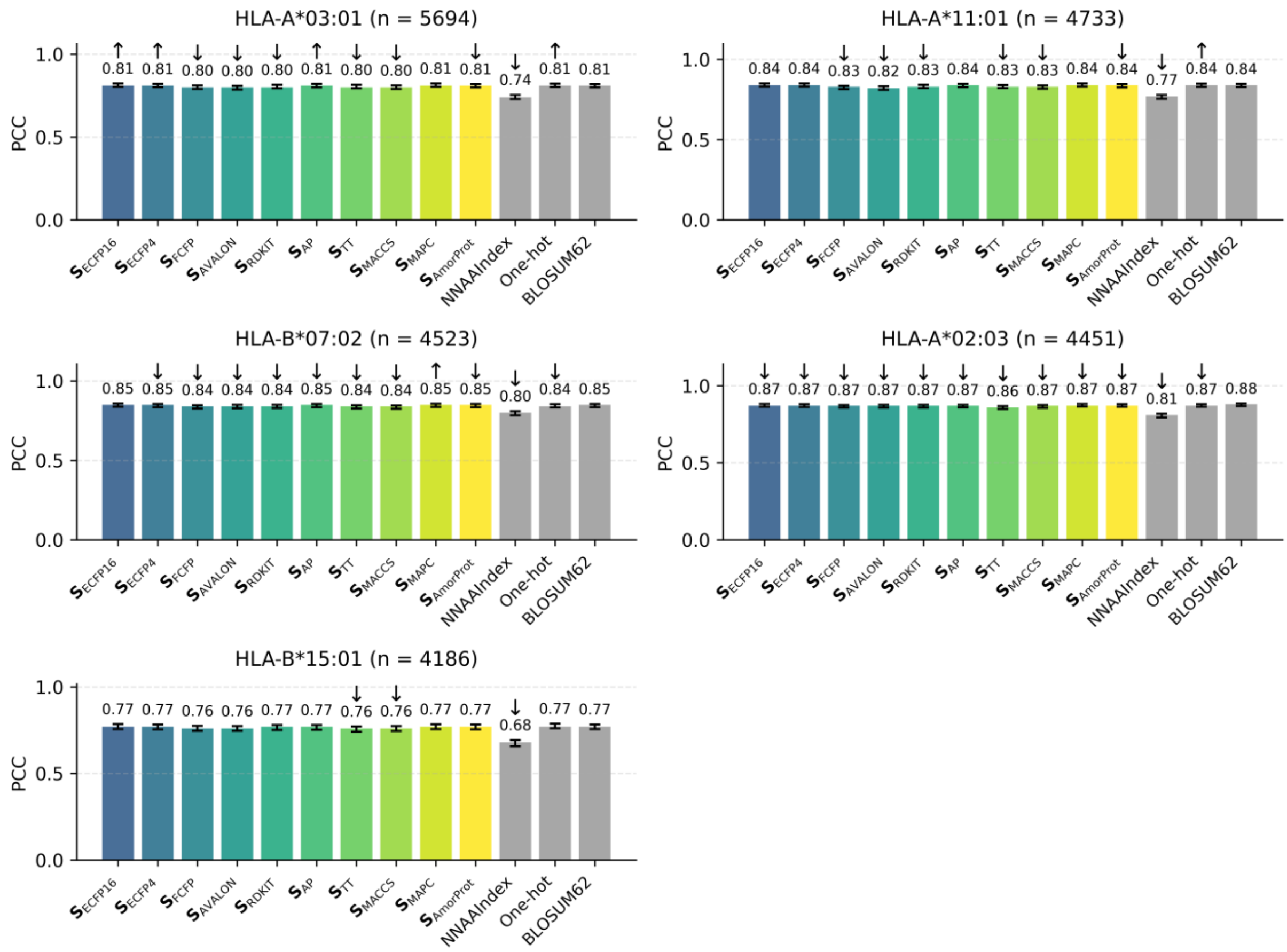

Supplementary Figure 15. PCC performance of similarity-based fingerprints for MHC class I binding prediction. PCC values from out-of-fold predictions for NAA-based peptides binding to selected HLA-A and HLA-B alleles. Each subplot shows results for one allele with sample size indicated in the title. Gray bars represent sequence-based encodings; coloured bars represent similarity-based fingerprints. Upward arrows indicate significantly better performance than BLOSUM62; downward arrows indicate significantly worse performance than BLOSUM62; absence of arrows indicates no significant difference from BLOSUM62 (paired Wilcoxon tests on per-peptide squared errors, FDR < 0.05, Benjamini-Hochberg correction for 12 pairwise comparisons per allele). Error bars report the 2.5th-97.5th percentiles of the bootstrap distribution (10,000 resamples).

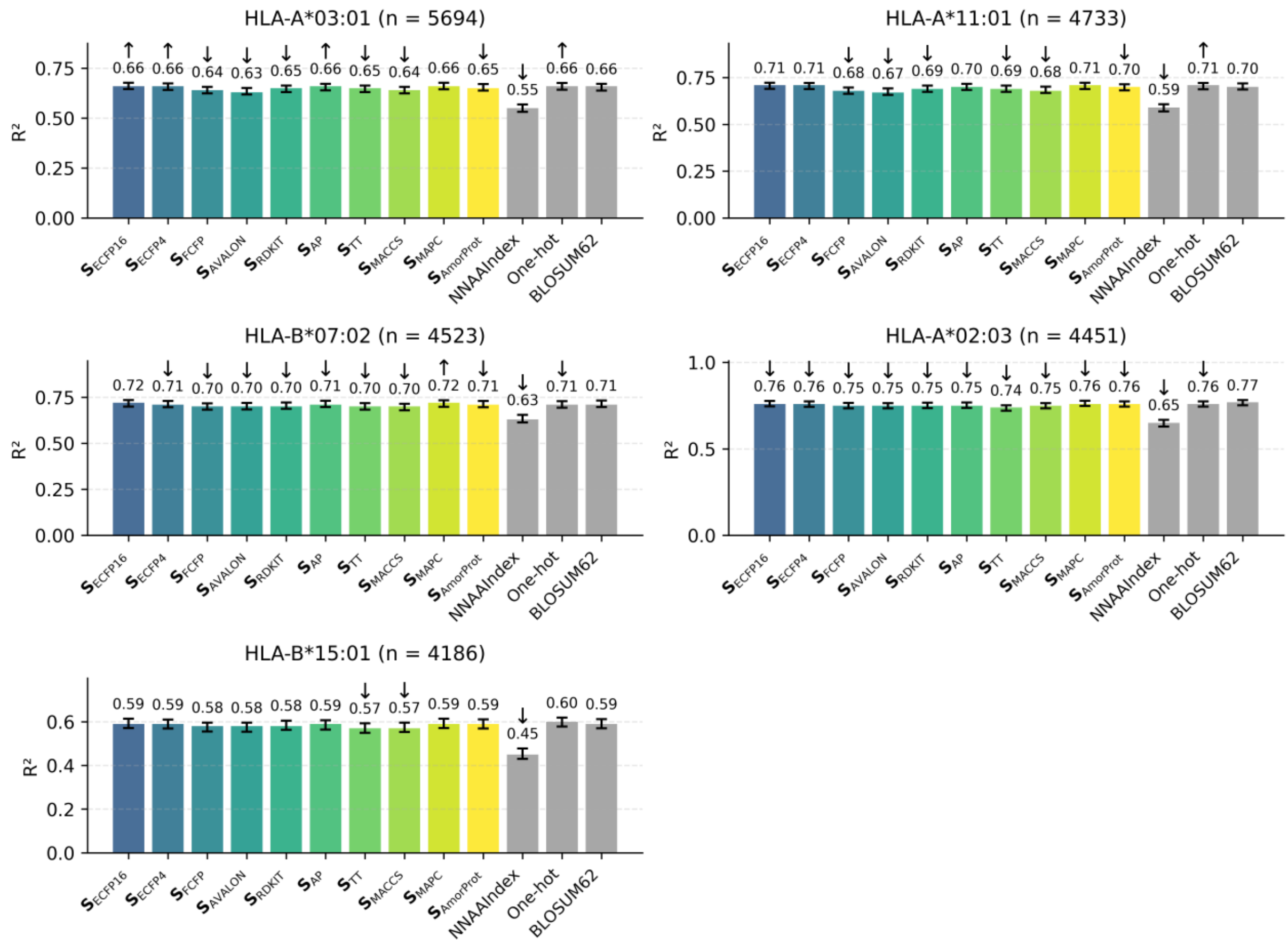

Supplementary Figure 16. R^2 performance of similarity-based fingerprints for MHC class I binding prediction. R^2 values from out-of-fold predictions for NAA-based peptides binding to selected HLA-A and HLA-B alleles. Each subplot shows results for one allele with sample size indicated in the title. Gray bars represent sequence-based encodings; coloured bars represent similarity-based fingerprints. Upward arrows indicate significantly better performance than BLOSUM62; downward arrows indicate significantly worse performance than BLOSUM62; absence of arrows indicates no significant difference from BLOSUM62 (paired Wilcoxon tests on per-peptide squared errors, FDR < 0.05, Benjamini-Hochberg correction for 12 pairwise comparisons per allele). Error bars report the 2.5th-97.5th percentiles of the bootstrap distribution (10,000 resamples).

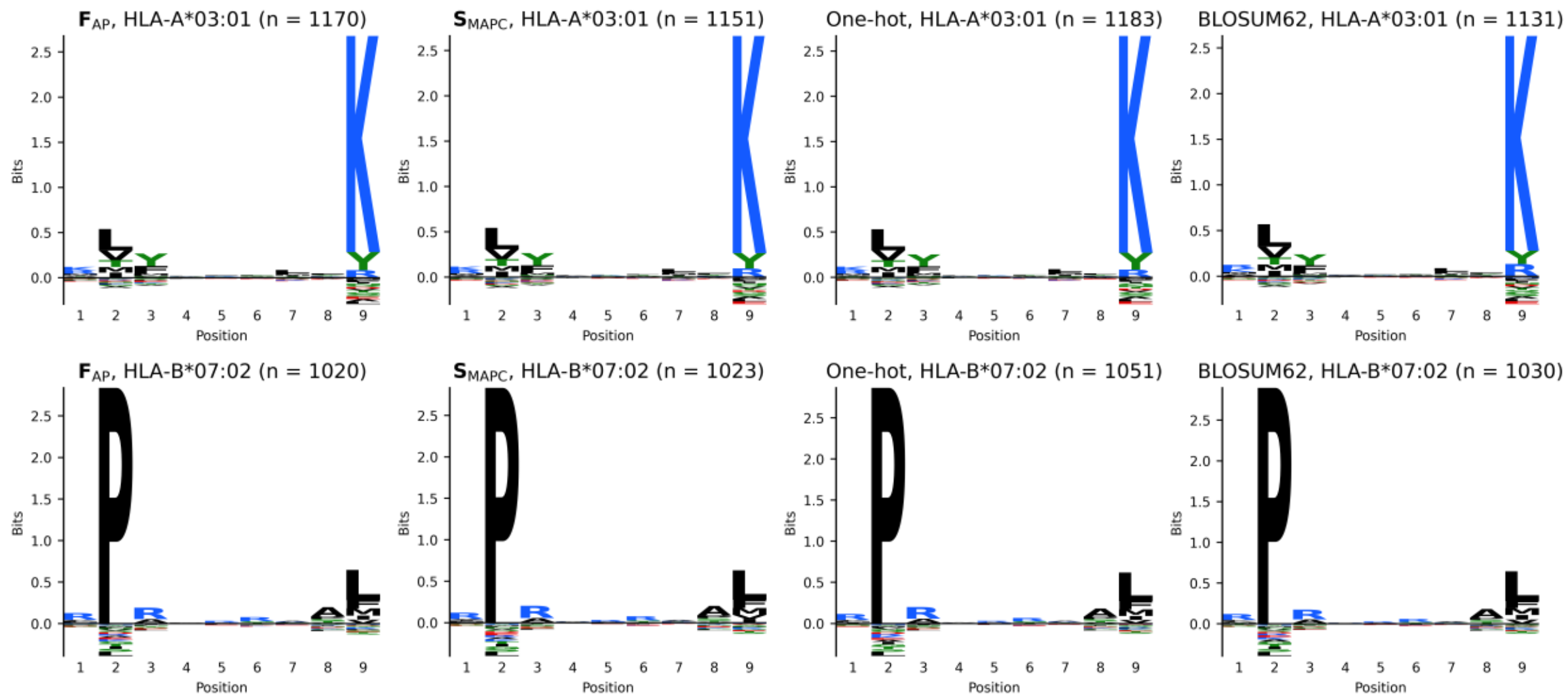

Supplementary Figure 17. Predicted binding motifs for MHC class I alleles. Sequence logos generated from out-of-fold predictions for peptides predicted as binders ($ba_{score} \geq 0.5$), considering $\mathbf{F}_{AP}$, $\mathbf{S}_{MAPC}$, one-hot and BLOSUM62. Logos display amino acid frequencies at each position, with letter height proportional to information content.

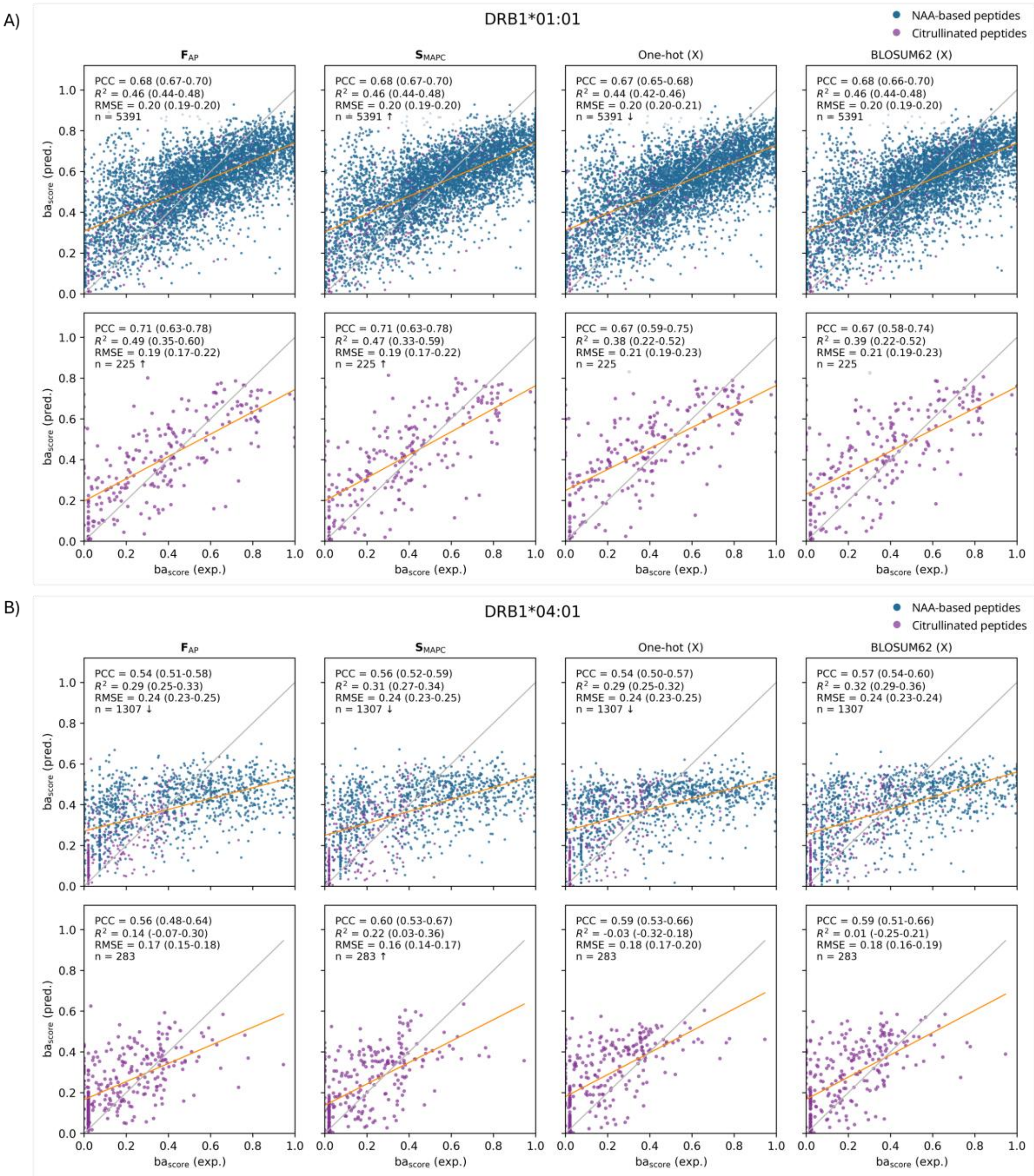

Supplementary Figure 18. Prediction performance for citrullinated peptides with models trained on NAA-based and citrullinated peptides, considering citrulline encoded as 'X' for sequence-based encodings. Binding affinity predictions for (**A**) DRB1*01:01 and (**B**) DRB1*04:01 using models trained using both NAA-based and citrullinated peptides. In each subplot, the first row shows out-of-fold predictions on both NAA-based and citrullinated peptides, while the second row shows predictions on citrullinated peptides only. Blue dots: NAA-based peptides; purple dots: citrullinated peptides. Yellow line: fitted regression; gray line: identity line. Each panel shows results for four encodings ($\mathbf{F}_{AP}$, $\mathbf{S}_{MAPC}$, one-hot, BLOSUM62) with PCC, R^2 and RMSE performance metrics, including the 2.5th and 97.5th percentiles of the bootstrap distribution (10,000 resamples). Upward arrows indicate significantly better performance than BLOSUM62 with citrulline encoded as 'X'; downward arrows indicate significantly worse performance than BLOSUM62 (X); absence of arrows indicates no significant difference (paired Wilcoxon tests on per-peptide squared errors, FDR < 0.05, Benjamini-Hochberg correction for 3 pairwise comparisons per allele). n indicates the number of predicted samples.

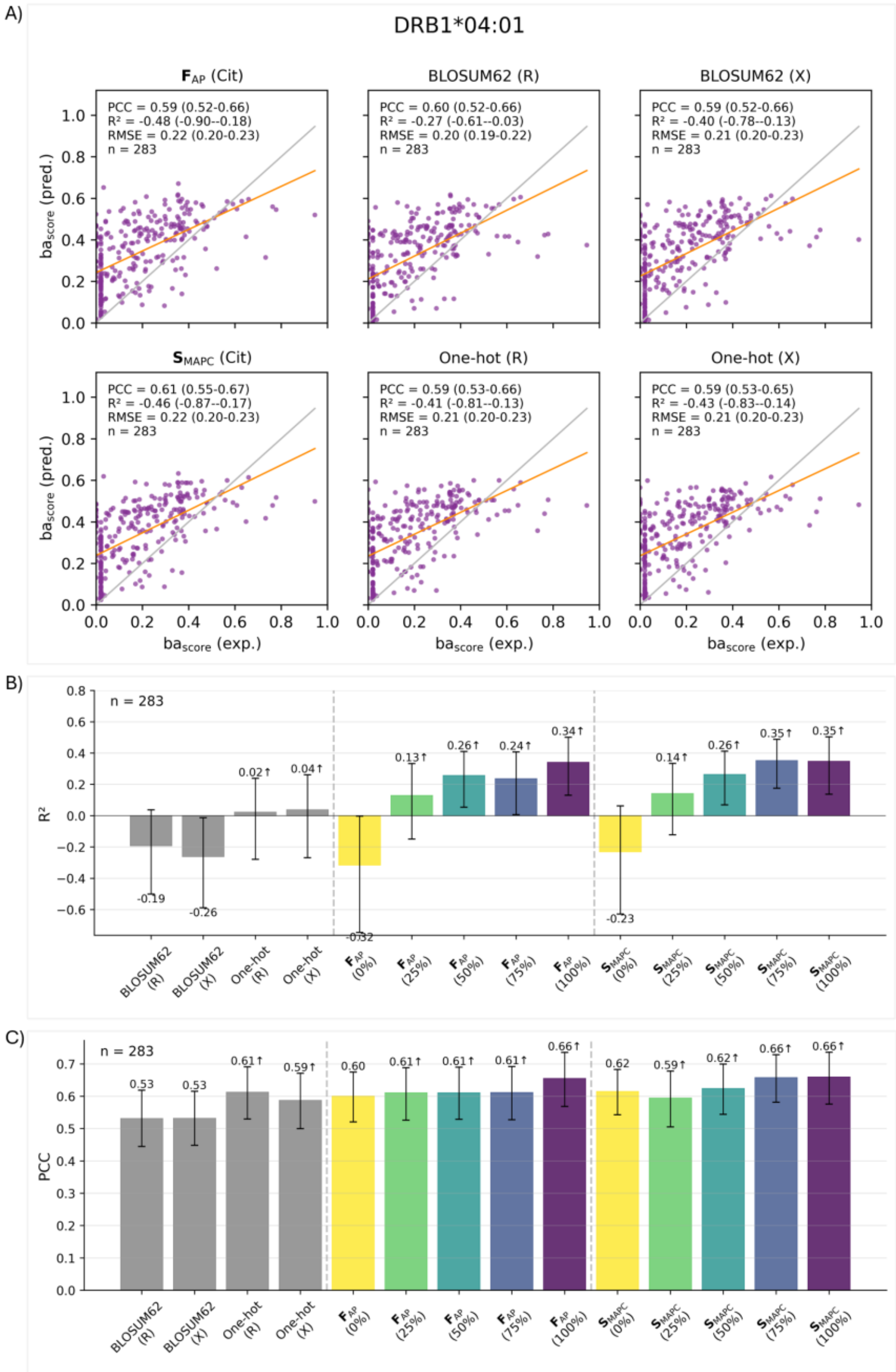

Supplementary Figure 19. Impact of training data composition on prediction performance for citrullinated peptides (DRB1*04:01). (**A**) Prediction performance on citrullinated peptides using models trained exclusively on NAA-based peptides. Yellow line: fitted regression; gray line: identity. Four encodings are compared: $\mathbf{F}_{AP}$ and $\mathbf{S}_{MAPC}$ encode citrulline via chemical fingerprints; one-hot and BLOSUM62 encode citrulline as 'X' (zero vector) or 'R' (arginine). PCC, R^2 and RMSE performance metrics, including the 2.5th and 97.5th percentiles of the bootstrap distribution (10,000 resamples), are reported. (**B**) R^2 and (**C**) PCC as a function of citrullinated peptide proportion in training data (0%, 25%, 50%, 75%, 100%). Models were evaluated using 5-fold cross-validation. Gray bars: BLOSUM62 and one-hot encodings trained only on NAA-based peptides, with citrulline encoded as 'X' or 'R'; coloured bars: $\mathbf{F}_{AP}$ and $\mathbf{S}_{MAPC}$ performance at each training composition. Error bars report the 2.5th-97.5th percentiles of the bootstrap distribution (10,000 resamples). Upward arrows indicate significantly better performance than BLOSUM62 with citrulline encoded as arginine (R); downward arrows indicate significantly worse performance than BLOSUM62 (R); absence of arrows indicates no significant difference (paired Wilcoxon tests on per-peptide squared errors, FDR < 0.05, Benjamini-Hochberg correction for 13 pairwise comparisons per allele).

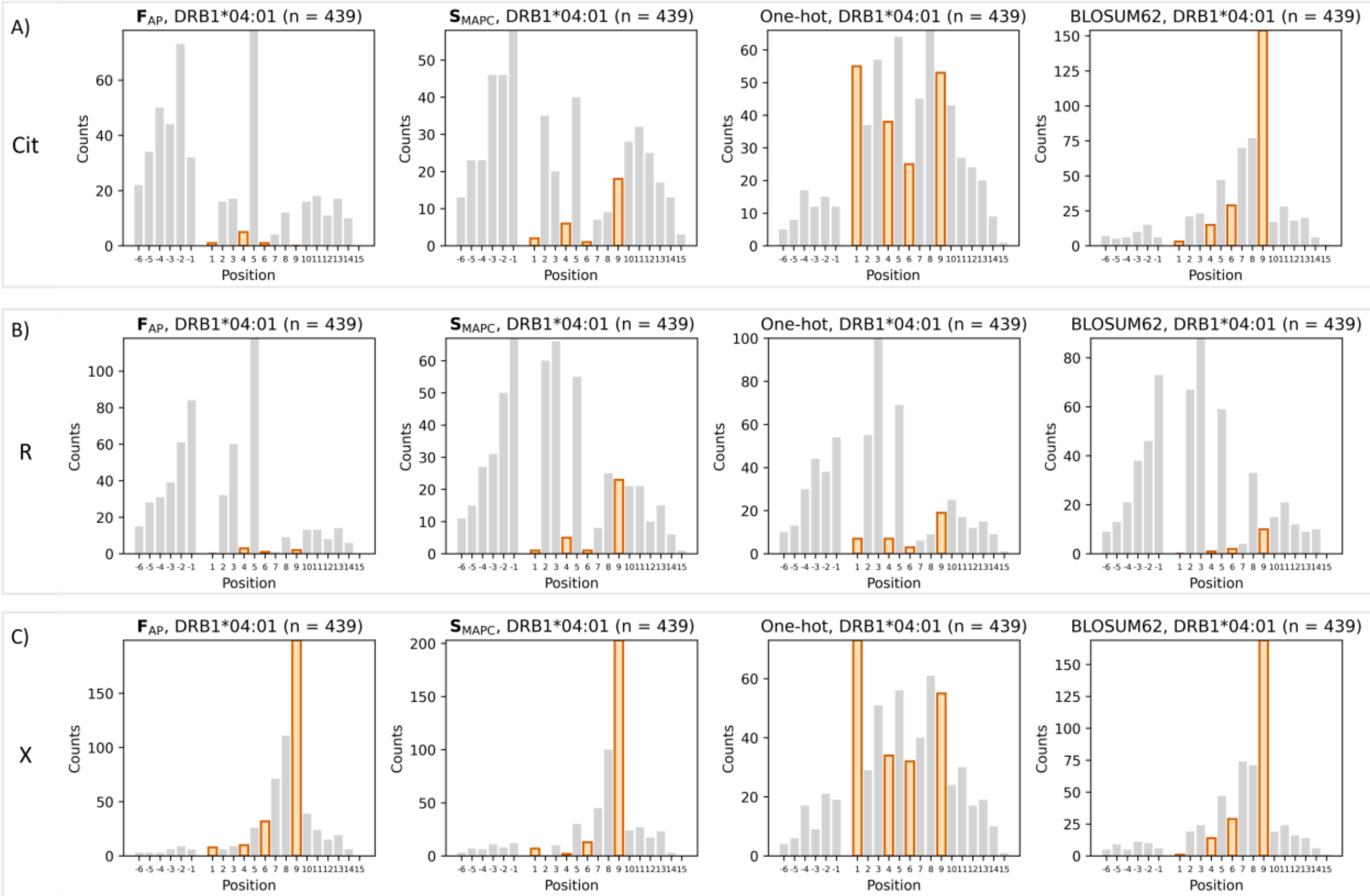

Supplementary Figure 20. Citrulline positional frequency in predicted binders for DRB1*04:01 under different encoding strategies. Positional occurrence of citrulline in top 1% predicted binders (n = 439) from citrullinated peptides (Rebak et al. (2)), comparing predictions from models trained with citrulline encoded as: (**A**) citrulline (chemical fingerprint for $\mathbf{F}_{AP}$ and $\mathbf{S}_{MAPC}$ and zero-vector for one-hot and BLOSUM62), (**B**) arginine (natural analogue), or (**C**) 'X' (unknown residue). X-axis shows position relative to the 9-mer binding core: positions 1-9 indicate core positions, negative numbers indicate N-terminal flanking positions, and numbers >9 indicate C-terminal flanking positions. Orange bars highlight frequency at anchor positions (P1, P4, P6, P9); gray bars show frequency at all other positions.

Supplementary Table 1. Hyperparameter search space for Optuna optimization of MLP, XGBoost and SVM models. MLP was implemented using PyTorch; XGBoost and SVM were implemented using the XGBoost Python package and scikit-learn, respectively. Hyperparameters were optimized with Optuna (20 trials), considering pruning and minimizing MSE on cross-validation sets. Early stopping was applied for XGBoost and MLP models.

Model	Hyperparameter	Search Space
**MLP**	hidden layer dimension	[8, 16, 32, 64] (categorical)
	dropout rate	[0.3, 0.7] (uniform float)
	learning rate	[10^{-4}, 10^{-2}] (log-uniform float)
	weight decay	[10^{-5}, 10^{-2}] (log-uniform float)
**XGBoost**	n_estimators	[50, 300] with step 25 (integer)
	max_depth	[2, 6] (integer)
	min_child_weight	[3, 10] (integer)
	learning_rate	[10^{-2}, 10^{-1}] (log-uniform float)
	subsample	[0.5, 0.9] (uniform float)
	colsample_bytree	[0.5, 0.9] (uniform float)
	reg_alpha (L1)	[0.0, 10.0] (uniform float)
	reg_lambda (L2)	[1.0, 10.0] (uniform float)
**SVM**	C	[10^{-1}, 10^{1}] (log-uniform float)
	epsilon	[10^{-3}, 5×10^{-1}] (log-uniform float)
	kernel	[rbf, linear] (categorical)
	gamma	[scale, auto] (categorical), optimized only when kernel = 'rbf'

References

1. Nielsen M, Lund O. Nn-Align. An Artificial Neural Network-Based Alignment Algorithm for Mhc Class Ii Peptide Binding Prediction. *BMC Bioinformatics* (2009) 10:296. doi: 10.1186/1471-2105-10-296.
2. Rebak AS, Hendriks IA, Elsborg JD, Buch-Larsen SC, Nielsen CH, Terslev L, et al. A Quantitative and Site-Specific Atlas of the Citrullinome Reveals Widespread Existence of Citrullination and Insights into Padi4 Substrates. *Nat Struct Mol Biol* (2024) 31(6):977-95. doi: 10.1038/s41594-024-01214-9.
